# Research on surface crack segmentation and quantitative degradation assessment in alpine meadows based on UAV imagery and the YOLO-AMSC model

**DOI:** 10.3389/fpls.2026.1895244

**Published:** 2026-08-21

**Authors:** Lihui Ma, Haili Zhu, Benfeng Li, Yuechen Wu, Pengkai Xu, Yaqi Yan, Guorong Li, Yabin Liu

**Affiliations:** 1 College of Geological Engineering, Qinghai University, Xining, China; 2 Laboratory of Cenozoic Resources and Environment on the Northern Margin of the Qinghai-Tibet Plateau, Xining, China; 3 Academy of Animal and Veterinary Sciences, Qinghai University, Xining, China; 4 School of Civil Engineering and Water Resources, Qinghai University, Xining, China

**Keywords:** cracks in the mattic epipedon of alpine meadows, degradation threshold, image segmentation, skeletal geometric constraints, YOLO-AMSC

## Abstract

Cracks in the mattic epipedon of alpine meadows are a key morphological indicator of ecosystem degradation. However, the accurate extraction of these minute and irregular crack features remains a significant challenge in complex natural environments characterized by vegetation cover, drastic fluctuations in light intensity, and soil texture interference. To address these challenges, a novel deep learning network, YOLO-AMSC, is proposed for the high-precision segmentation and morphological quantification of cracks in the mattic epipedon of alpine meadows under complex field environments. A Spatial to Depth Attention Fusion (SDAF-Block) module is proposed to preserve fine-grained spatial details of minute cracks without information loss and to reconstruct their broken topological continuity. Simultaneously, a Mixed Local Channel Attention (MLCA) mechanism is introduced to adaptively enhance crack textures while suppressing background noise. Furthermore, a hierarchical focused loss function, termed HFP-IoU (Hierarchical Focal-Penalty IoU), is designed to impose strict geometric constraints on the elongated crack boundaries via a non-monotonic focusing mechanism. The experimental results demonstrate that YOLO-AMSC achieves superior segmentation performance, improving mAP50 by 4.27%, 2.96%, 2.43%, 4.58%, 8.86%, 12.51%, 22.03%, 55.77%, and 52.19% compared with YOLOv5n-seg, YOLOv10n-seg, YOLO-hyper-seg, YOLOv12n-seg, SOLOv2, SparseInst, DeepCrack, CrackFormer-II, and CrackSegDiff, respectively. Using the high-fidelity segmentation masks from this model and a skeleton-based geometric constraint method for crack width estimation, the relative error relative to field measurements is less than 10%. Employing a system dynamics framework, this study quantitatively determines the topological width thresholds of 0.6 cm and 3.1 cm, which signify a sudden increase in connectivity and indicate an accelerated degradation process of alpine meadows. This method directly links pixel-level image analysis with regional early warning systems, delivering a cost-effective, non-destructive digital framework for dynamic ecological monitoring.

## 1 Introduction

The Qinghai-Tibet Plateau is highly sensitive to global climate change, with its alpine meadows acting as a critical ecosystem component indispensable for biodiversity conservation, climate regulation, water retention, and soil erosion control (Zhou et al., 2023). However, global warming, overgrazing, and intensified freeze-thaw and wet-dry cycles have severely compromised the stability of the mattic epipedon in alpine meadows (Cao et al., 2013). The initiation and propagation of fissures in the mattic epipedon of alpine meadows are primary physical indicators of degradation; the geometry of these fissures evolves from linear to dendritic, ultimately generating intricate, irregular polygonal networks (Miehe et al., 2019; Wu et al., 2024b). These cracks disrupt the continuity of the mattic epipedon, exacerbating soil moisture and nutrient loss while acting as primary conduits for erosion, subsidence, and patchy degradation. Consequently, they serve as critical morphological indicators of the irreversible transition toward severe alpine meadow degradation (Niu et al., 2019; Li et al., 2025). Accurately extracting crack geometry and quantifying crack width and distribution are crucial for establishing early diagnostic thresholds for alpine meadow degradation.

Accurate feature extraction remains the primary bottleneck in crack detection, prompting researchers across diverse disciplines to develop various algorithms. Traditional methods are typically categorized into three main types: thresholding (Li et al., 2023b), edge detection (Lv et al., 2022) and region growing algorithms (Li et al., 2026a). However, these methods are relatively inefficient and rely heavily on extensive image pre-processing, which inherently limits their adaptability and generalization capabilities for complex crack detection (Cuevas et al., 2010). The development of crack datasets and deep learning techniques has greatly advanced crack research, especially by driving the evolution of segmentation model architectures (Song et al., 2025). Consequently, these models achieve superior detection accuracy, efficiency, and generalization. Specifically, CNN-based object detection frameworks are broadly categorized into two-stage and one-stage architectures. In crack detection tasks, representative two-stage models include R-CNN (He et al., 2022), Faster R-CNN (Liu et al., 2023a), and U-Net (Zhu et al., 2026a). While highly accurate, these models suffer from architectural complexity and immense computational overhead, severely hindering their real-time deployment in practical scenarios.

Conversely, single-stage architectures, most notably the YOLO (You Only Look Once) series, have emerged as the dominant paradigm, gaining immense traction by successfully balancing accuracy with real-time processing efficiency (Bai et al., 2026; Lu et al., 2025; Xu et al., 2025). The YOLO series has evolved to integrate instance segmentation while retaining high inference speeds, advancing from bounding-box detection to pixel-level precision. Specifically, YOLOv8-seg frameworks offer a robust solution for accurately segmenting cracks in the mattic epipedon of alpine meadows, achieving an optimal trade-off between computational efficiency and accuracy (Zhu and Zhou, 2025; Tian et al., 2025). While demonstrating excellent performance on man-made structures like roads (Du et al., 2025), bridges (Su et al., 2025; Liu et al., 2023b), and tunnels (Duan et al., 2024), its direct application to unstructured natural environments, particularly for segmenting cracks in the mattic epipedon of alpine meadows, still faces significant challenges. Specifically, vegetation occlusion, soil texture interference, and dynamic illumination severely obscure the features of cracks in the mattic epipedon of alpine meadows. Consequently, standard models struggle to generalize in such complex scenarios, making targeted optimizations of the neural network mechanisms essential for robust and accurate detection.

In high-resolution remote-sensing imagery, adaptive feature enhancement and cross-scale feature fusion improve the representation of densely distributed small objects by integrating shallow spatial-detail features with deep semantic information (Zheng et al., 2023). However, these methods mainly focus on cracks on artificial structural surfaces, where the backgrounds usually show stronger rigidity and relatively stable texture patterns. In contrast, cracks in the mattic epipedon of alpine meadows develop in a highly heterogeneous natural environment with intensive biological activity and are jointly disturbed by roots, dry grass, bare soil particles, shadows, and discontinuous vegetation cover. Therefore, the key challenge of this study is not only to enlarge the receptive field or perform local pixel calibration, but also to preserve the fine-grained crack topology during downsampling, recover discontinuous crack skeletons under occlusion, and suppress pseudo-linear texture interference from vegetation and soil under the constraints of real-time UAV monitoring.

To address these challenges, this paper proposes YOLO-AMSC, an improved segmentation model designed for the high-precision segmentation and morphological analysis of cracks in the mattic epipedon of alpine meadows. A novel Space to Depth Attention Fusion (SDAF) block is integrated between the backbone and neck networks to capture long-range spatial dependencies and enhance pixel-level attention. This architecture enables the precise extraction of continuous skeletons and edge structures of cracks in the mattic epipedon of alpine meadows amidst complex vegetative backgrounds. To mitigate the loss of fine details in deep neural networks, traditional strided convolutions are replaced with a space-to-depth transformation. This preserves fine-grained spatial information during downsampling, significantly enhancing the detection of subtle cracks in the mattic epipedon of alpine meadows. Furthermore, integrating the Mixed Local Channel Attention (MLCA) module refines channel-wise feature representations, thereby bolstering the model’s discriminative power against complex soil backgrounds. To date, deep learning applications for crack detection in complex natural environments remain limited. Notably, systematic studies that directly translate the high-precision segmentation of cracks in the mattic epipedon of alpine meadows into quantitative degradation assessments are rare. To bridge this gap, we propose an automated degradation evaluation framework based on our segmentation results. Unlike traditional qualitative approaches, we introduce a skeleton-geometry-constrained algorithm to calculate the exact physical width of cracks in the mattic epipedon of alpine meadows. This establishes a quantitative link between crack morphology and the degree of degradation, facilitating end-to-end monitoring. Ultimately, our method offers an efficient, low-cost, and non-destructive tool for assessing alpine meadow ecosystems.

## 2 Materials and methods

### 2.1 Image acquisition and dataset construction

**Figure 1:**
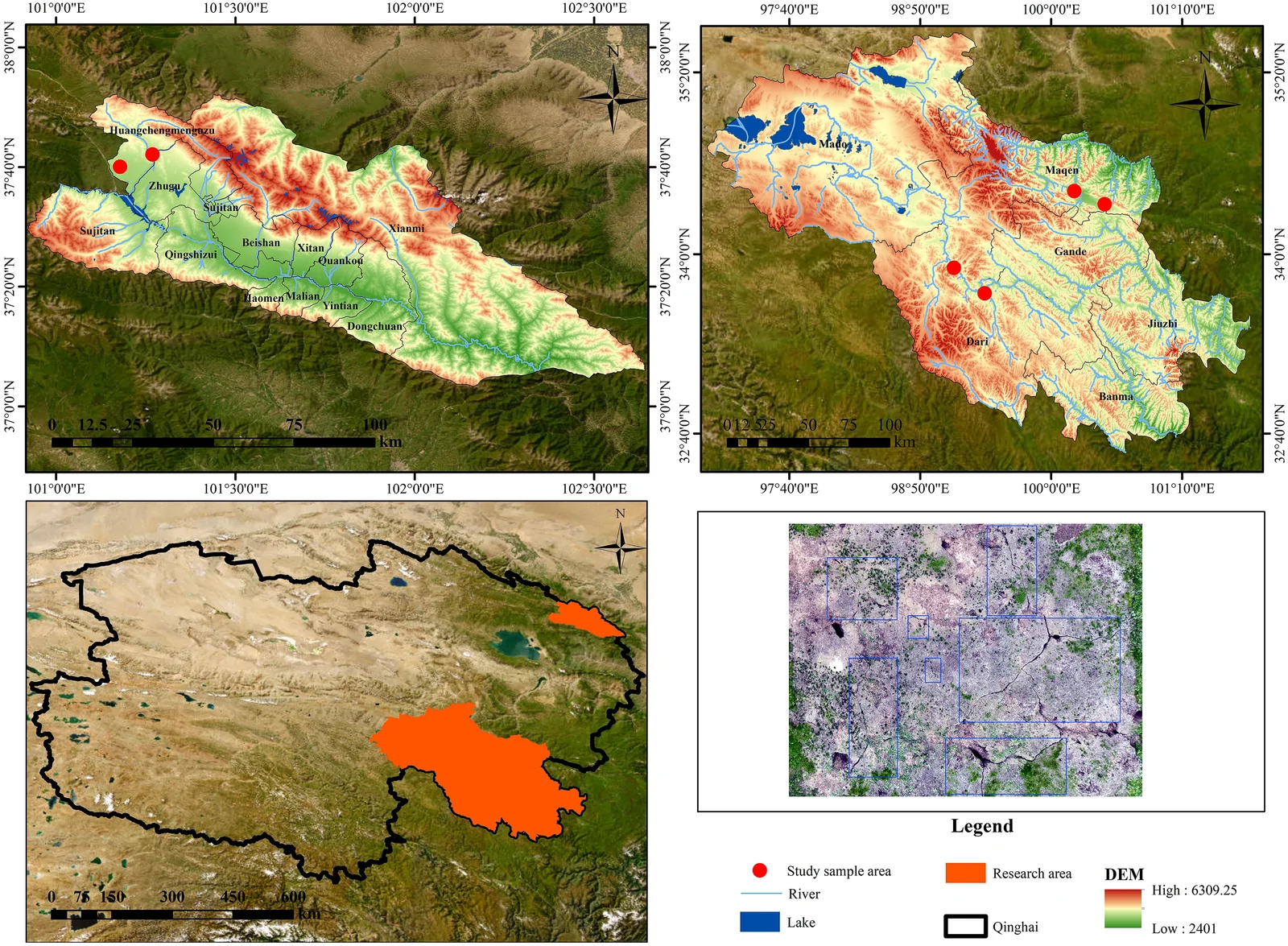
Location of the study area.

The formation of cracks in the mattic epipedon of alpine meadows is closely driven by seasonal freeze-thaw cycles, typically occurring from late October to March. As temperatures rise, these cracks gradually close in early May, with their numbers diminishing significantly by the peak growing season (Wang et al., 2020). Previous studies have shown that, in grassland ecosystems of the Qinghai–Tibet Plateau, grassland cracks mostly occur in the surface layer of alpine meadows, whereas other grassland types generally do not exhibit typical and continuous crack development in the mattic epipedon. The distribution, formation mechanisms, and ecological effects of alpine grassland cracks are closely associated with the degradation process of alpine meadows (Niu, 2020). Therefore, the site selection in this study focused on alpine meadow cracks as the specific research object. As shown in **Figure 1**, field surveys of surface crack development were conducted in November and June, and three field regions were selected to reflect the spatial heterogeneity of alpine meadows. The degradation status of these regions was assessed according to the Assessment Standard for Degradation Status of Alpine Mattic Epipedons (DB63/T 1413–2015) (Qinghai Provincial Bureau of Quality and Technical Supervision, 2015), and the regions were classified into mild, moderate, and severe degradation stages. Within these regions, six typical sites of 150 × 150 m were delineated for UAV data acquisition, aiming to cover the main crack development stages and representative morphological characteristics within the study area.

To ensure the spatial scale accuracy of UAV imagery and the reliability of subsequent crack width calculation, four ground control points were placed at the four corners of each 150 m × 150 m typical site for spatial calibration during UAV image mosaicking and orthorectification. The flight altitude and camera parameters were mainly used for flight planning and theoretical ground sampling distance (GSD) estimation, whereas the actual pixel size used for model training images and crack width conversion was derived from the orthomosaic calibrated by the ground control points. To ensure high-precision detection of cracks in the mattic epipedon of alpine meadows, UAV operations adhered to the settings of Wu et al (Wu et al., 2024a). For data acquisition, the UAV operated at a constant altitude of 15 m and a flight speed of 2 m/s, with longitudinal and transversal image overlaps configured to 80% and 70%, respectively.

**Figure 2:**
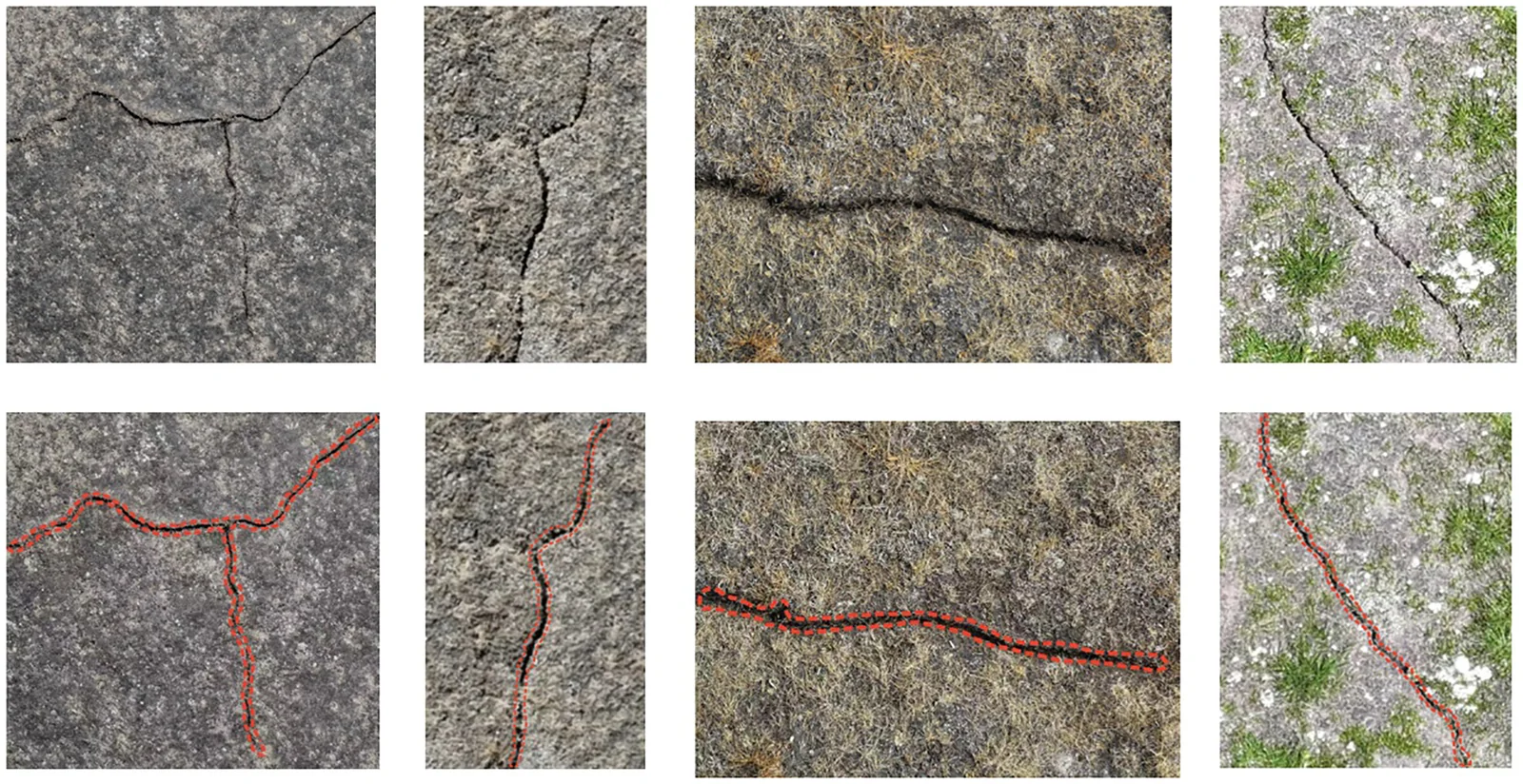
Data and annotated images of cracks in the mattic epipedon of alpine meadows.

Based on field sites in Menyuan, Dari, and Maqin counties, Qinghai Province, an original dataset of cracks in the mattic epipedon of alpine meadows was constructed. A total of 5,305 UAV images were acquired and manually annotated using LabelMe. To ensure annotation consistency, only regions with clear boundaries, continuous dark depressions, or identifiable crack geometry were labeled as cracks, whereas ambiguous regions easily confused with bare soil textures, roots, dry grass, or shadows were excluded. For vegetation-occluded cracks, only the visible segments were annotated, without artificially connecting occluded gaps. The annotations were manually reviewed by overlaying them on the original images, and missing labels, incorrect labels, boundary offsets, and disputed samples were corrected. The final dataset was first grouped according to the image acquisition site and UAV flight route. Images belonging to the same site–flight-route group were assigned exclusively to a single subset, ensuring that spatially adjacent or visually similar UAV images did not appear simultaneously in the training, validation, and test sets. All image groups from the six sites were then pooled and randomly partitioned at the group level into training, validation, and test sets at a ratio of 8:1:1. This strategy was adopted to minimize spatial information leakage and provide a more reliable evaluation of model generalization. Representative images and their corresponding annotations are shown in **Figure 2**.

### 2.2 Method overview

**Figure 3:**
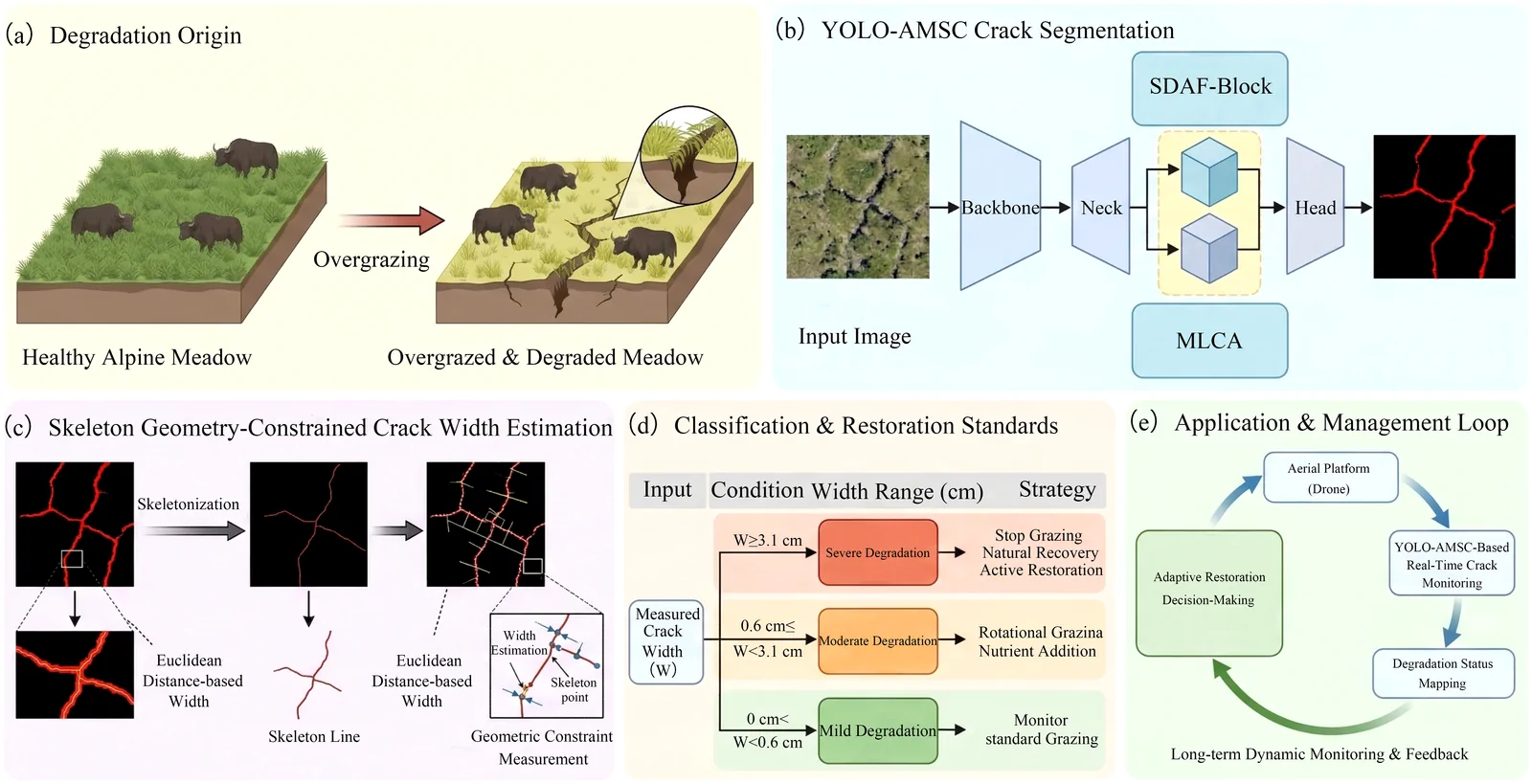
Workflow for crack segmentation and degradation assessment in the mattic epipedon of alpine meadows using YOLO-AMSC: **(a)** degradation origin; **(b)** crack segmentation using YOLO-AMSC; **(c)** crack-width estimation based on skeleton geometric constraints; **(d)** degradation classification and restoration strategies; and **(e)** application and adaptive-management feedback loop.

The subtle cracks in the mattic epipedon of alpine meadows exhibit complex topological structures and are frequently obscured by vegetation, complicating their accurate extraction and quantitative assessment via traditional methods. To address these challenges, this study proposes an improved model, YOLO-AMSC, which integrates the SDAF-Block and MLCA mechanisms. Replacing standard strided convolutions with SPDConv effectively mitigates feature loss during downsampling, thereby preserving fine-grained spatial information. Furthermore, the proposed SDAF-Block leverages the long-range dependency modeling of CAFM to reconstruct the continuous skeleton structure of cracks in the mattic epipedon of alpine meadows. Combined with PixelAttention-CGA, which dynamically suppresses complex background noise, the model achieves significantly enhanced edge discrimination in low-contrast environments. The MLCA mechanism enhances the discriminative representation of cracks in the mattic epipedon of alpine meadows through multi-level feature interaction and channel-adaptive recalibration. Consequently, it effectively suppresses meadow background interferences, such as vegetation texture, bare soil particles, and shadows. Furthermore, an end-to-end quantification framework based on skeletal geometric constraints is developed, bridging ‘image perception’ with ‘ecological diagnosis.’ Ultimately, this provides robust technical support for the dynamic regional-scale monitoring of ecosystems. The overall workflow of the proposed framework is shown in **Figure 3**.

### 2.3 Construction of the YOLO-AMSC network

#### 2.3.1 YOLO-AMSC overall structure

**Figure 4:**
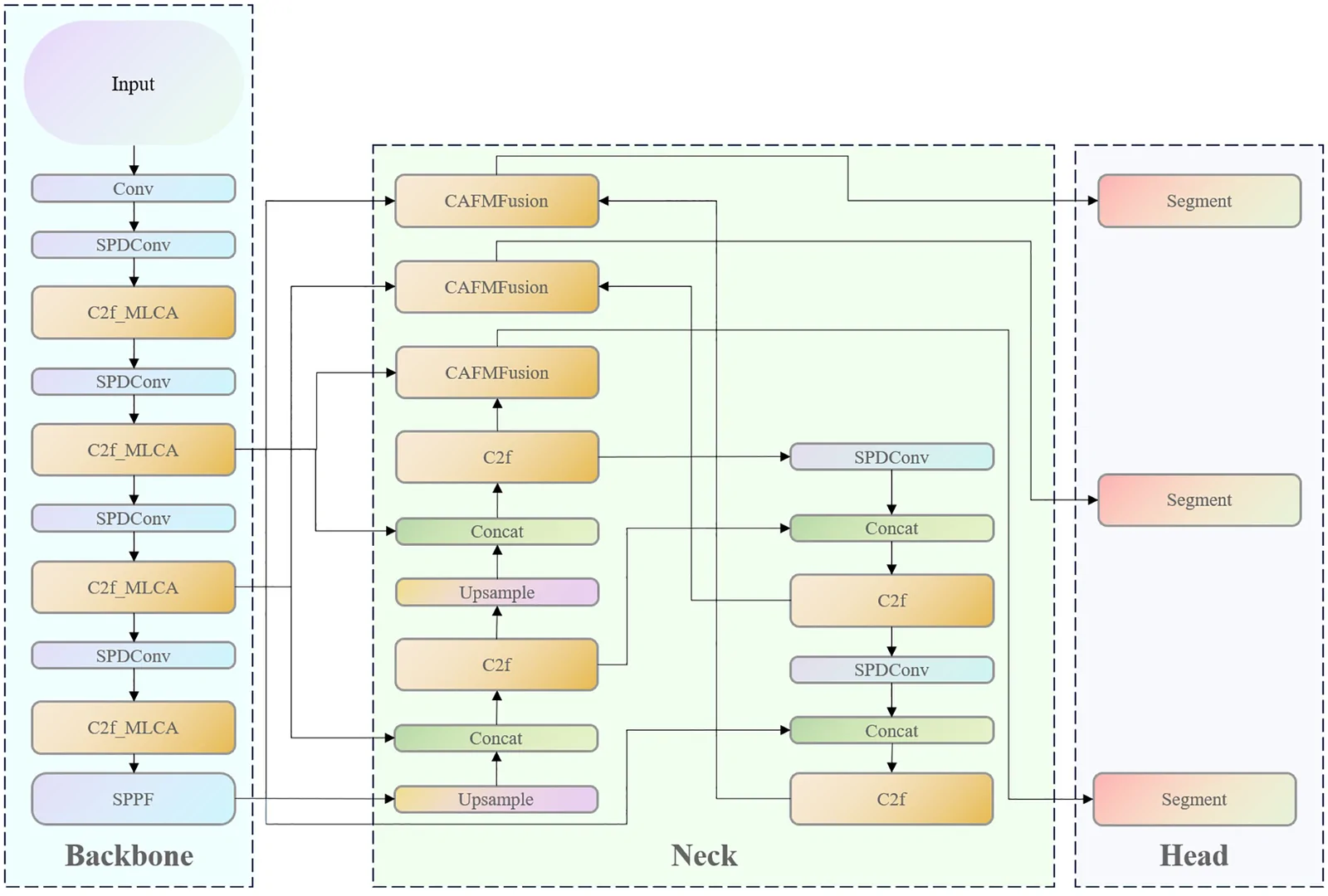
YOLO-AMSC network architecture.

The YOLOv8 series exhibits exceptional capabilities in multi-scale object detection. Consequently, YOLOv8n-seg was chosen as the baseline model to achieve an optimal trade-off between computational overhead and segmentation precision. To specifically address the distinct morphological features of cracks in the mattic epipedon of alpine meadows, substantial architectural enhancements were introduced to the backbone and neck networks. The YOLO-AMSC model, depicted in **Figure 4**, ensures the highly accurate segmentation of minor cracks against complex backgrounds.

#### 2.3.2 SDAF-Block network architecture

**Figure 5:**
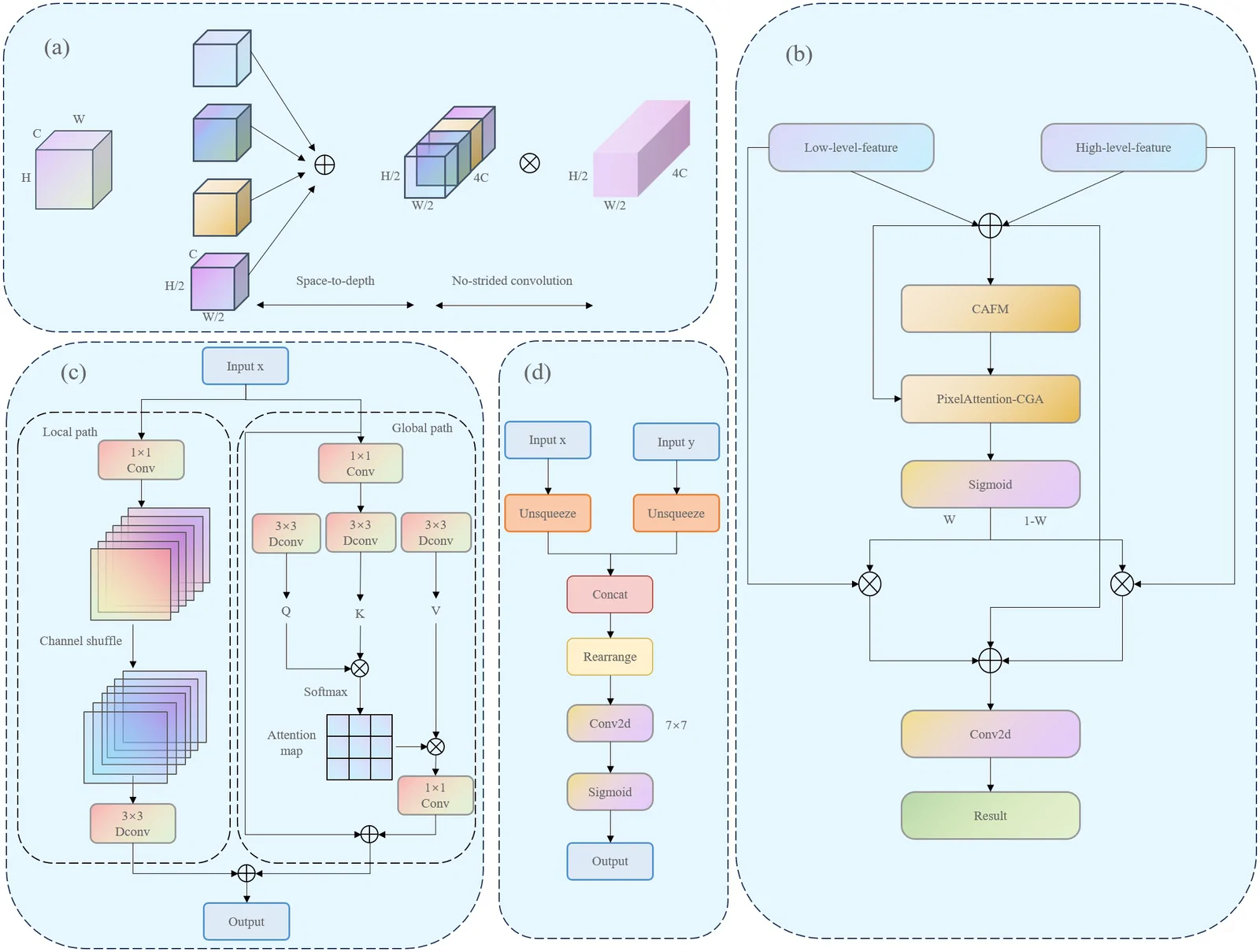
SDAF-Block network architecture: **(a)** SPDConv network architecture, **(b)** Fusion network architecture, **(c)** CAFM network architecture, **(d)** PixelAttention-CGA network architecture.

In the conventional YOLOv8 architecture, multi-scale feature extraction and downsampling often lead to pixel degradation and skeletal discontinuities, presenting significant obstacles for the quantitative analysis of cracks in the mattic epipedon of alpine meadows. Consequently, we introduce the Space to Depth Attention Fusion (SDAF) module, whose detailed architecture is shown in **Figure 5**. Engineered with SPDConv, CAFM, PixelAttention-CGA, and an adaptive fusion strategy, this module retains low-level spatial features while reconstructing high-level semantics, maximizing segmentation accuracy against complex background interference.

The SPDConv-based downsampling method for micro-cracks avoids using strides greater than 1. SPDConv conducts alternate slicing on the input feature map with a fixed stride of step=2 to generate four sub-tensors in the spatial dimension, which are then concatenated in the channel dimension. This process reduces the spatial resolution by half and doubles the number of channels, converting the dimensions from B*C*H*W to B*4C* H/2*W/2. Subsequently, a 3×3 convolution is applied for feature mapping. This reorganization in the spatial dimension ensures maximum preservation of edge details and topological structures of micro-cracks, providing a robust foundation for precise pixel-level width measurements. Because of the intertwined root systems of surface vegetation, cracks in the mattic epipedon of alpine meadows are frequently occluded or visually fragmented. To address the resulting disconnection during segmentation, a topology-aware, context-sensitive CAFM module is integrated. Initially, input features are upsampled and projected into a higher-dimensional space. Subsequently, 3×3 depthwise separable convolutions extract local joint features across spatial and channel dimensions, accurately capturing irregular edge variations. Concurrently, following feature restructuring, a multi-head self-attention mechanism is employed to compute pixel-wise correlation weights across the global receptive field. By leveraging these long-range dependencies, the model effectively infers the topological continuity of cracks across regions obscured by dry grass or shadows, thereby resolving segmentation mask inconsistencies. The visual similarity between the soil texture and the shadows within cracks in the mattic epipedon of alpine meadows inherently leads to blurred boundaries. To achieve precise width estimation, the PixelAttention-CGA module is proposed. This approach integrates original fused features with CAFM-derived context via channel-wise concatenation. To capture extensive spatial contexts, a 7 ×7 convolution featuring a broad receptive field is applied alongside a 2D convolution with reflective padding. Subsequently, a sigmoid-activated pixel-level spatial attention map is generated to amplify responses along the crack skeleton and attenuate high-frequency noise in non-target areas, thereby refining the final segmentation boundaries.

The SDAF-Block adopts the CAFMFusion adaptive aggregation strategy, where x denotes the crack feature stream and y represents the background context stream. First, CAFMFusion integrates these two streams to produce fused features. Then, CAFM is utilized to extract primary global topological features, which are subsequently fed into PixelAttention-CGA to compute a pixel-level weight map. The final feature aggregation process is expressed in Equation 1 as follows:

(1) $$\text{Result}={\text{Conv}}_{1\times 1}\left(initial+pattn2x+(1-pattn2)y\right)$$

This mechanism establishes a dynamic feature balance via soft attention weights. In regions with high detection confidence, the network prioritizes the primary feature stream x for cracks in the mattic epipedon of alpine meadows. Conversely, in transitional areas like crack edges or vegetation-obscured zones, the model adaptively incorporates the contextual feature stream y to enhance inference. This physics-guided fusion strategy enables the YOLO-AMSC model to generate segmentation masks with preserved morphology and distinct boundaries despite complex natural disturbances. Consequently, it lays a solid foundation for subsequent detailed assessments of mild, moderate, and severe degradation levels guided by skeletal geometric constraints and width thresholds.

#### 2.3.3 MLCA network architecture

**Figure 6:**
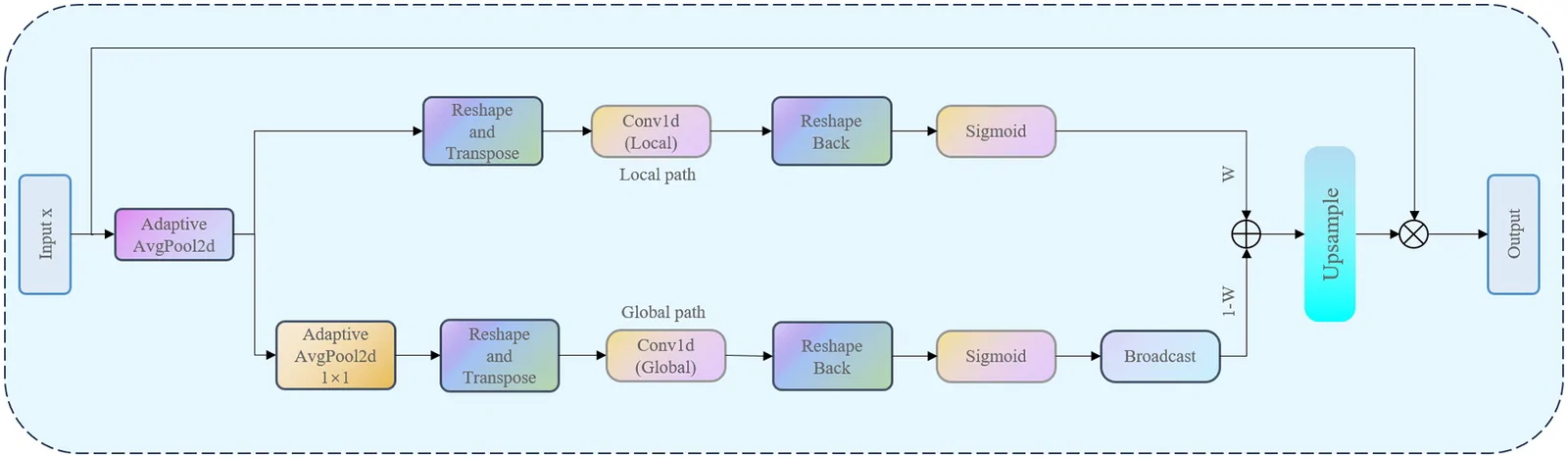
MLCA network architecture.

In complex natural environments, standard convolutional operations struggle to distinguish high-frequency background noise from the channel-wise features of cracks in the mattic epipedon of alpine meadows. Consequently, models frequently misclassify linear shadows as cracks or fail to detect them entirely. To address prior limitations and enhance the model’s sensitivity to the critical texture features of cracks in the mattic epipedon of alpine meadows, the C2f_MLCA module is utilized. While retaining the standard YOLOv8n cross-stage partial network architecture, it effectively upgrades the internal bottleneck to a Bottleneck_MLCA unit integrating Mixed Local Channel Attention (MLCA). The MLCA network architecture is shown in **Figure 6**. The MLCA mechanism merges local and global features by applying global average pooling (GAP) for global channel statistics and local convolutions for neighborhood spatial correlations. This hybrid aggregation enables the network to discern orientation while preserving fine edge textures. Subsequently, the module models and weights channel dependencies through 1D convolutions, facilitating robust cross-channel interactions. The corresponding channel-weighting formulation is given in Equation 2:

(2) $$\omega =\sigma \left(Conv_{1\text{D}}(GAP(X)+Local(X))\right)$$

A sigmoid activation function is employed to modulate feature contributions. These weights signify the relative importance of individual channels for the segmentation of cracks in the mattic epipedon of alpine meadows. By multiplying the generated weights with the original feature map channel-wise, the model adaptively suppresses weaker background responses, such as large vegetation patches or uniform soil regions, while significantly enhancing channels with strong crack textures.

### 2.4 Loss function

Cracks in the mattic epipedon of alpine meadows often exhibit a highly elongated morphology, which challenges the metric sensitivity of conventional Intersection over Union (IoU) computations. Minor pixel-level displacements along the shorter edge of a predicted bounding box can drastically degrade the IoU value, potentially reducing it to zero. This hypersensitivity deprives the model of meaningful gradient feedback during early training phases. Furthermore, the standard 1-IoU loss struggles to differentiate between complete non-overlap and marginal overlap caused by slight deviations, lacking the adequate geometric constraints necessary for the precise regression of elongated objects (Wang et al., 2025a; Le Jeune and Mokraoui, 2023). To mitigate this challenge, this paper introduces a novel loss function termed HFP-IoU (Hierarchical Focal-Penalty IoU). This algorithm modifies the gradient surface of the regression loss by incorporating a hierarchical geometric penalty term alongside a non-monotonic focal modulation mechanism.

#### 2.4.1 Corner alignment-based normalized geometric penalty term

Unlike conventional IoU, which focuses solely on area overlap, HFP-IoU first computes the absolute differences between the top-left and bottom-right corners of the predicted B_pred_ and ground-truth B_gt_ bounding boxes. These differences are then normalized by the object scale. The corner-deviation terms and the normalized geometric penalty term are formulated in Equations 3–7 as follows:

(3) $$dw_{1}=\left|x_{1}^{p}-x_{1}^{\text{gt}}\right|$$

(4) $$dw_{2}=\left|x_{2}^{p}-x_{2}^{\text{gt}}\right|$$

(5) $$d_{h1}=\left|y_{1}^{p}-y_{1}^{\text{gt}}\right|$$

(6) $$d_{h2}=\left|y_{2}^{p}-y_{2}^{\text{gt}}\right|$$

(7) $$P=\frac{1}{4}\left(\frac{dw_{1}+dw_{2}}{\left|w_{\text{gt}}\right|}+\frac{dh_{1}+dh_{2}}{\left|h_{\text{gt}}\right|}\right)$$

Within the normalization constraint, the penalty term achieves scale adaptability by incorporating the bounding box dimensions as the denominator. For extremely narrow cracks in the mattic epipedon of alpine meadows, this small denominator dictates that even minor displacements along the shorter edge incur a substantial penalty. This mechanism enforces rigorous regression along the minor axis, effectively resolving the localization challenges of slender features. Furthermore, unlike center-point distances, corner deviations directly reflect boundary alignment, thereby enhancing the precision of the segmentation mask at the crack extremities.

#### 2.4.2 Focal modulation mechanism based on non-monotonicity

To tackle the imbalance in samples between easy and challenging classes, this study utilizes a dynamic weighting factor derived from a Gaussian function. Introducing an intermediate variable *q* and a focal variable *x*, where Λ is a hyperparameter, the intermediate variables and the resulting HFP-IoU output are expressed in Equations 8 and 9, respectively:

(8) $$q=e^{-P},\;x=\Lambda \cdot q=\Lambda \cdot e^{-P}$$

(9) $$HFP-IoU=3\cdot x\cdot e^{-x^{2}}\cdot \left(IoU-e^{-P^{2}}+1\right)$$

The formula $f(x)=3xe^{-x^{2}}$ defines the function as the gain coefficient. In dynamic gradient weighting, when there is a low overlap between the predicted bounding box and the ground-truth bounding box when P is large and x is small, the gain coefficient enters an increasing phase. Consequently, the gradients are amplified appropriately at this juncture, facilitating rapid convergence of the model. As the overlap is gradually optimized towards the ‘critical region’ with moderate P, the gain coefficient peaks, leading the model to heavily prioritize challenging samples. Conversely, with extremely high overlap where P nears 0 and x nears Λ, the gain coefficient decreases gradually. Simultaneously, to mitigate overfitting, the non-monotonic focusing mechanism prevents the model from overly emphasizing easy samples in the latter training stages, thus averting outliers from inducing excessive gradients that may distort the feature space.

For the detection of cracks in the mattic epipedon of alpine meadows, the normalized penalty term P significantly amplifies error signals along the width dimension. This enables the model to discern minute width discrepancies around the critical degradation thresholds of 0.6 cm and 3.1 cm, thereby fundamentally enhancing width regression accuracy. Incorporating PIoU, defined by a base term $IoU-e^{-P^{2}}+1$ combined with corner constraints, optimizes bounding box alignment with the intricate curvatures of cracks in the mattic epipedon of alpine meadows. Such alignment substantially reduces the risk of incorporating background noise from adjacent vegetation. In complex high-altitude environments characterized by target-mimicking shadows, this mechanism efficiently neutralizes disturbances from simple negative samples. Therefore, it promotes the rapid recognition of authentic targets, expediting model convergence and elevating the ultimate mAP performance.

### 2.5 A novel crack width measurement method based on skeleton geometric constraints

Existing methods for quantifying crack width, including morphological erosion, pixel accumulation statistics, and enhancement algorithms based on super-resolution reconstruction, essentially all rely on counting discrete pixel grids (Yamaguchi et al., 2008; Orton et al., 2025; Che et al., 2018). However, due to the inherent discretized raster nature of digital images, accurately representing the physical width along the crack’s normal vector remains challenging, especially for complex curved targets. This study introduces a width computation approach based on skeletal geometric constraints. By deriving the topological skeleton and transforming discrete image signals into continuous geometric curve functions, this method facilitates precise width calculations at each skeleton node along its orthogonal normal. Unlike conventional pixel-based statistics, this approach mitigates grid-induced cumulative errors, optimizing alignment with the actual morphology and enhancing the quantification precision for irregular cracks in the mattic epipedon of alpine meadows.

YOLO-AMSC first extracts the skeleton and boundaries of cracks in the mattic epipedon of alpine meadows. Based on three-point interpolation, pixel coordinates are fitted to a skeleton curve function to derive normal line equations for each node. By approximating crack edges as linear segments, we determine their intersections with the normal lines. The width at each point is then calculated via the distance formula. This procedure is applied to all nodes to determine the corresponding crack widths across the entire target area.

#### 2.5.1 Pixel size calibration

For calculated widths of cracks in the mattic epipedon of alpine meadows to reflect real-world dimensions, image pixel sizes must be precisely aligned with the actual scale. Thus, pixel calibration is performed as a mandatory step before width estimation (Liu et al., 2025). The pixel side length can be determined using Equation 10:

(10) $$\eta =\frac{B}{f}\sqrt{\frac{S_{c}}{R_{I}}}$$

In this equation, the actual pixel edge length *η* (mm) is determined by combining the flight distance (*B*, mm) with the camera hardware parameters (*f*, mm; *S_c_* mm^2^). Calibrating this spatial metric subsequently enables the precise localization of each pixel’s centroid within the 2D planar coordinate system.

Following single-pixel width calibration, pixels of the upper boundary, skeleton, and lower boundary of cracks in the mattic epipedon of alpine meadows are sequentially ordered along the crack strike. Utilizing a 3×3 rectangular kernel, the algorithm identifies the start and end points before traversing and sorting pixels along each curve. For the upper edge, skeleton, and lower edge, the total pixel counts are denoted as L, N, and M, respectively. Accordingly, each pixel is assigned an index from 1 to L, 1 to N, or 1 to M, corresponding to its relative position along the specific curve’s orientation.

#### 2.5.2 Calculation of the midpoint normal line equation

**Figure 7:**
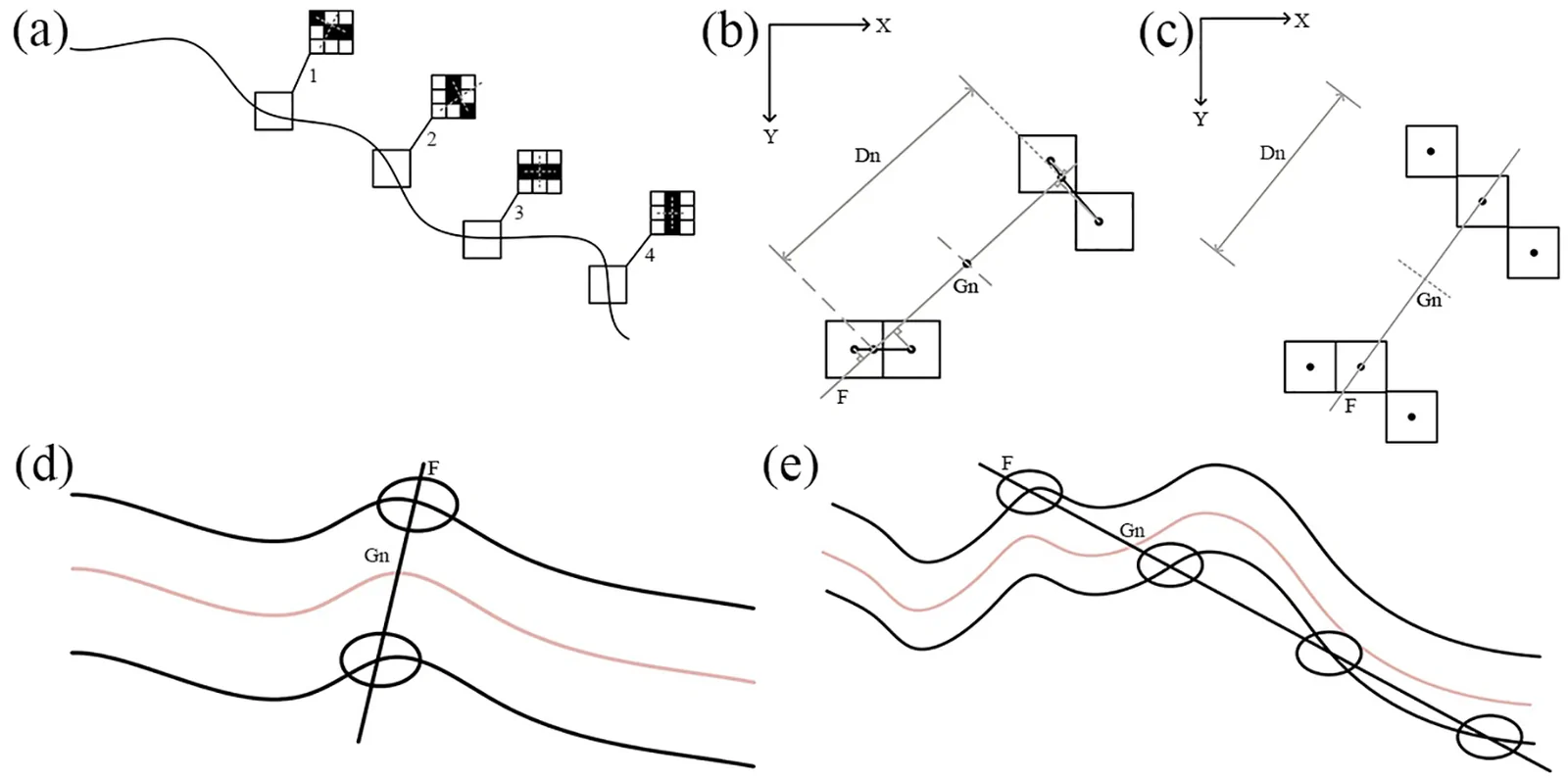
Method for calculating crack widths in structural frames: **(a)** Schematic diagram of crack orientation, **(b)** Schematic diagram of crack calculation at two intersections, **(c)** Schematic diagram of crack calculation at three intersections, **(d)** Schematic diagram of a single-intersection crack curve, **(e)** Schematic diagram of a multi-intersection crack curve.

The local skeleton curvature and its corresponding midpoint normal are estimated by applying quadratic interpolation y = ax^2^ + bx + c to the Cartesian positions of three consecutive medial pixels: G_n-1_, G_n_, and G_n+1_ (n ∈ [2, N-1]). Subsequently, the precise normal equation at the central node is formulated by classifying the local micro-geometry into four distinct structural scenarios represented by Equations 11–14. Under the specific spatial constraints of distinct nodal coordinates (x_n-1_ ≠ x_n_ ≠ x_n+1_; y_n-1_ ≠y_n+1_), as depicted in **Figure 7a**, the normal equation F for the focal pixel G_n_(x_n_, y_n_, n) is mathematically derived from its local gradient k_n_, which is acquired by evaluating the first derivative of the fitted polynomial curve.

(11) $$x+k_{n}y-\left(x_{n}+k_{n}y_{n}\right)=0$$

As illustrated by position 2 in **Figure 7a**, for micro-structural arrangements where standard spatial interpolation fails due to overlapping coordinate constraints, an axial transposition from the XOY to the YOX domain is utilized. Evaluating the polynomial derivative within this reversed plane yields a transitional normal equation, F^-1^. Reversing this specific mathematical mapping seamlessly provides the true orthogonal equation F at the medial node G_n_(x_n_, y_n_, n) within the original Cartesian space.

(12) $$k_{x}^{\prime }+y-\left(k_{n}^{\prime }x_{n}+y_{n}\right)=0$$

Based on the topological features at position 3 in **Figure 7a**, satisfying the prerequisite x_n-1_ ≠ x_n+1_ results in a strictly horizontal tangent at pixel G_n_. Because this specific tangent possesses a zero gradient, the subsequent normal equation F is expressed as:

(13) $$y=y_{n}$$

Subject to the topological condition y_n+1_ = y_n-1_ shown at position 4 in **Figure 7a**, the medial curve develops a purely vertical tangent possessing an infinite slope at G_n_, leading to the following definition for the normal vector F:

(14) $$x=x_{n}$$

#### 2.5.3 Determination of intersection point coordinates

The crack boundary curves are linearized within local intervals flanking each normal line. The intersection coordinates between these fitted upper and lower boundary lines and the normal equation are then determined to facilitate precise width estimation.

In scenarios characterized by tortuous crack trajectories, the normal line may yield redundant intersections with edge curves, as shown in (**Figures 7d, e**). A localized search mechanism is therefore necessitated to isolate the nearest pixel points. Following directional pixel ordering, the search boundaries are established based on the absolute disparities between the terminal indices L and M of the boundary curves and the corresponding index N of the skeleton curve. Such a constrained search ensures maximum fidelity in pinpointing the boundary intersections of cracks in the mattic epipedon of alpine meadows.

Following the transversal projections illustrated in **Figures 7b, c**, boundary intersections along the upper margin are identified. A localized search interval, [n-i, n+i] (i_s_ = |N-L|), is subsequently established around these specific nodes. By applying orthogonal distance constraints, the perpendicular spatial deviation l_sn_ of each boundary pixel from the normal vector is sequentially evaluated.

With the orthogonal vector defined geometrically as Ax + By + C = 0, the perpendicular shift l_sn_ of surrounding margin pixels is quantified. Within the neighborhood interval [n-i_s_, n+i_s_], two opposing boundary pixels closest to the normal axis are identified, specifically S_sj_ exhibiting the minimal positive l_sn_ and S_k_ demonstrating the maximal negative l_sn_. Integrating these specific spatial nodes yields the localized linear function for the superior boundary, formulated in Equation 15 as follows:

(15) $$\left(y_{sj}-y_{sk}\right)\left(x-x_{sj}\right)-\left(x_{sj}-x_{sk}\right)\left(y-y_{sj}\right)=0$$

Illustrated by the structural projections in **Figure 7b** and **c**, the precise orthogonal node H_sn_ on the upper boundary is ascertained via a dual-condition protocol. Provided a specific pixel within the search interval registers an absolute zero deviation l_sn_ = 0, it is immediately designated as H_sn_. Alternatively, without a perfectly intersecting pixel, the exact spatial coordinates for H_sn_ are computed by algebraically intersecting the linear approximation of the local superior boundary with the established normal vector. Following the geometric procedures outlined in **Figures 7b, c**, spatial intersections along the lower crack margin are evaluated within the specific search interval [n-i_t_, n+i_t_] assuming i_t_=|N-M|. With the orthogonal normal standardized to Ax + By + C = 0, the perpendicular distance l_tn_ for each constituent lower boundary pixel is quantified to identify two opposing focal pixels: T_tj_ with the smallest positive l_tn_ and T_tk_ with the largest negative l_tn_. Unifying these specific boundary nodes mathematically reconstructs the local linear segment of the inferior margin, yielding the expression given in Equation 16:

**Figure 8:**
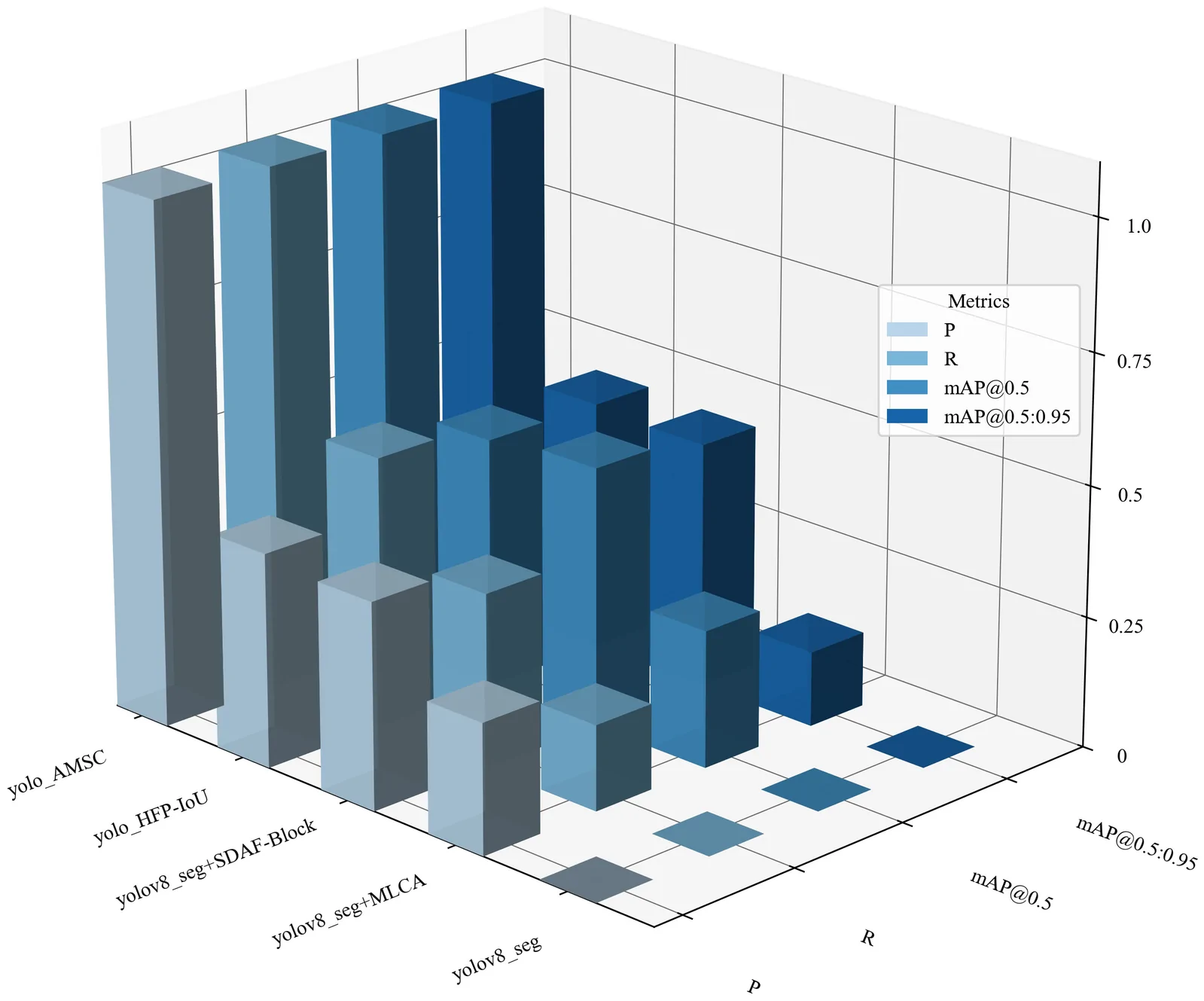
Comparison of performance in ablation experiments during the segmentation of cracks in the mattic epipedon of alpine meadows.

(16) $$\left(y_{tj}-y_{tk}\right)\left(x-x_{tj}\right)-\left(x_{tj}-x_{tk}\right)\left(y-y_{tj}\right)=0$$

Visualized via the structural projections in **Figures 7b, c**, relationship between mean acquiring the precise orthogonal node H_tn_ on the lower boundary necessitates evaluating two distinct spatial conditions. Provided a localized pixel registers an absolute zero deviation L_tn_=0, it is instantly classified as H_tn_. Alternatively, lacking a perfectly aligned pixel, the exact coordinates for H_tn_ are algebraically extracted by intersecting the derived normal vector with the localized linear trajectory of the lower edge.

#### 2.5.4 Calculation of the distance between intersection points

The structural width D_n_ at each specific medial pixel is derived by computing the perpendicular distance between the dual boundary intersections, as given in Equation 17 (Pan et al., 2023; Li et al., 2024). In this context, D_n_ defines the precise morphological width intersecting the transversal normal at a specific central pixel n. Replicating this geometric evaluation across all internal medial nodes, indexed from 2 to N-1, establishes a continuous measurement of the structural degradation along the primary axis.

(17) $$D_{n}=\sqrt{{\left(y_{tn}-y_{sn}\right)}^{2}+{\left(x_{tn}-x_{sn}\right)}^{2}}$$

## 3 Experimental setup

### 3.1 Test platform

The deep learning models in this study were executed on a workstation featuring an Intel Core i9-14900KF processor, 128 GB of RAM, and an NVIDIA GeForce RTX 5060 Ti graphics card, utilizing PyTorch 2.7.0 (CUDA 12.8). The model underwent training for 100 epochs with a batch size of 64. The same data augmentation settings were applied to all models, including HSV color-space adjustment, random translation, random scaling, horizontal flipping, Mosaic augmentation, RandAugment, and random erasing. To combat overfitting, the hyperparameters specified in **Table 1** were utilized.

**Table 1:** Hyperparameter configuration.

Hyperparameter	Value
Input size	640 × 640
Optimizer	SGD
Initial learning rate (lr0)	0.01
Final OneCycleLR learning rate (lrf)	0.01
Momentum	0.937
Weight decay	0.0005
workers	16
Random seed	0
Deterministic	True

### 3.2 Evaluation indicators

To guarantee the research methodology’s reliability and the experimental results’ validity, this study utilizes mean average precision (mAP), precision (P), and recall (R) as crucial performance evaluation metrics (Ding et al., 2023; Zang et al., 2026; Zhu et al., 2026b). The mathematical expressions for these metrics are specified in Equations 18–21 as follows:

(18) $$P=\frac{TP}{TP+FP}$$

(19) $$R=\frac{TP}{TP+FN}$$

(20) $$AP=\int_{0}^{1}P(R)\text{d}R$$

(21) $$mAP=\frac{\sum_{i=1}^{n}AP_{i}}{n}$$

In these metrics, TP refers to the number of crack instances accurately detected by the model; FP corresponds to background interferences, such as vegetation textures or shadows, erroneously classified as cracks; and FN represents actual meadow cracks omitted by the network.

## 4 Experimental results

### 4.1 Ablation experiments

This study validated the YOLO-AMSC network against the YOLOv8n-seg baseline, with comparison results detailed in **Table 2**. Integrating the MLCA mechanism improved the mAP50 to 63.52% without incurring additional computational overhead. This indicates that MLCA’s channel re-calibration strategy effectively suppresses background interferences, such as dry grass and bare soil, enhancing feature representation efficiently. The independent integration of the SDAF-Block module yielded a 1.69% increase in mAP50 and a 2.14% rise in recall, demonstrating its efficacy in feature preservation and its capacity to mitigate topological disruptions in cracks in the mattic epipedon of alpine meadows. Although FLOPs and parameter counts rose to 16.9G and 5.61M respectively, resulting in a marginal FPS decline, the YOLO-AMSC model maintained a speed of 218.52 FPS. This significantly exceeds the real-time monitoring threshold of 30 FPS required for drone-based or portable inspections (Luan et al., 2024; Liao and Juang, 2022). Transitioning to the HFP-IoU loss function yielded a 3.32% increase in recall over the baseline, with mAP50 reaching 64.26%. This validates the efficacy of the non-monotonic focusing mechanism without incurring additional inference latency. As the modules were progressively integrated, the model’s accuracy metrics exhibited a consistent upward trend. The final YOLO-AMSC model achieved 68.73% precision, 66.29% recall, and 66.03% mAP50. Notably, the absolute 6.43% surge in recall is of decisive importance for ecological applications; it ensures that the vast majority of subtle early-stage cracks in the mattic epipedon of alpine meadows are identified, providing a robust foundation for subsequent width quantification. In conclusion, YOLO-AMSC achieves optimal segmentation performance at a reasonable computational cost, constituting a practically valuable framework for ecosystem monitoring.

**Table 2:** Ablation experiments.

ID	Model	MLCA	SDAF-Block	HFP-IoU	P	R	mAP50	mAP50-95	Flops	Parameter	FPS
1	yolov8-seg	×	×	×	66.60%	59.86%	62.63%	23.91%	12	3.26	385.56
2	yolov8-seg	✓	×	×	67.13%	60.91%	63.52%	24.08%	12	3.26	363
3	yolov8-seg	×	✓	×	67.44%	62.00%	64.32%	24.47%	16.9	5.61	220.81
4	yolov8-seg	×	×	✓	67.47%	63.18%	64.26%	24.48%	12	3.26	387.11
5	yolov8-seg	✓	✓	×	67.33%	62.95%	64.72%	24.62%	16.9	5.61	217.61
6	yolov8-seg	×	✓	✓	66.63%	61.95%	64.55%	25.19%	16.9	5.61	221.63
7	yolov8-seg	✓	×	✓	67.88%	60.99%	63.26%	24.06%	12	3.26	371.66
8	yolo-AMSC	✓	✓	✓	68.73%	66.29%	66.03%	25.10%	16.9	5.61	218.52

As shown in **Figure 8**, YOLO-AMSC outperforms the YOLOv8n-seg baseline in precision, recall, mAP50, and mAP50–95. The higher recall indicates an improved ability to identify subtle cracks and reduce missed detections, while the simultaneous increase in precision suggests that this improvement is achieved without introducing excessive false positives. These gains demonstrate that the combined mechanisms enhance crack-feature representation, suppress interference from complex meadow backgrounds, and improve pixel-level localization and boundary delineation. Consequently, YOLO-AMSC provides more reliable segmentation of small and irregular cracks in the mattic epipedon of alpine meadows, supporting subsequent morphological quantification and degradation assessment.

### 4.2 Comparison experiments

**Figure 9:**
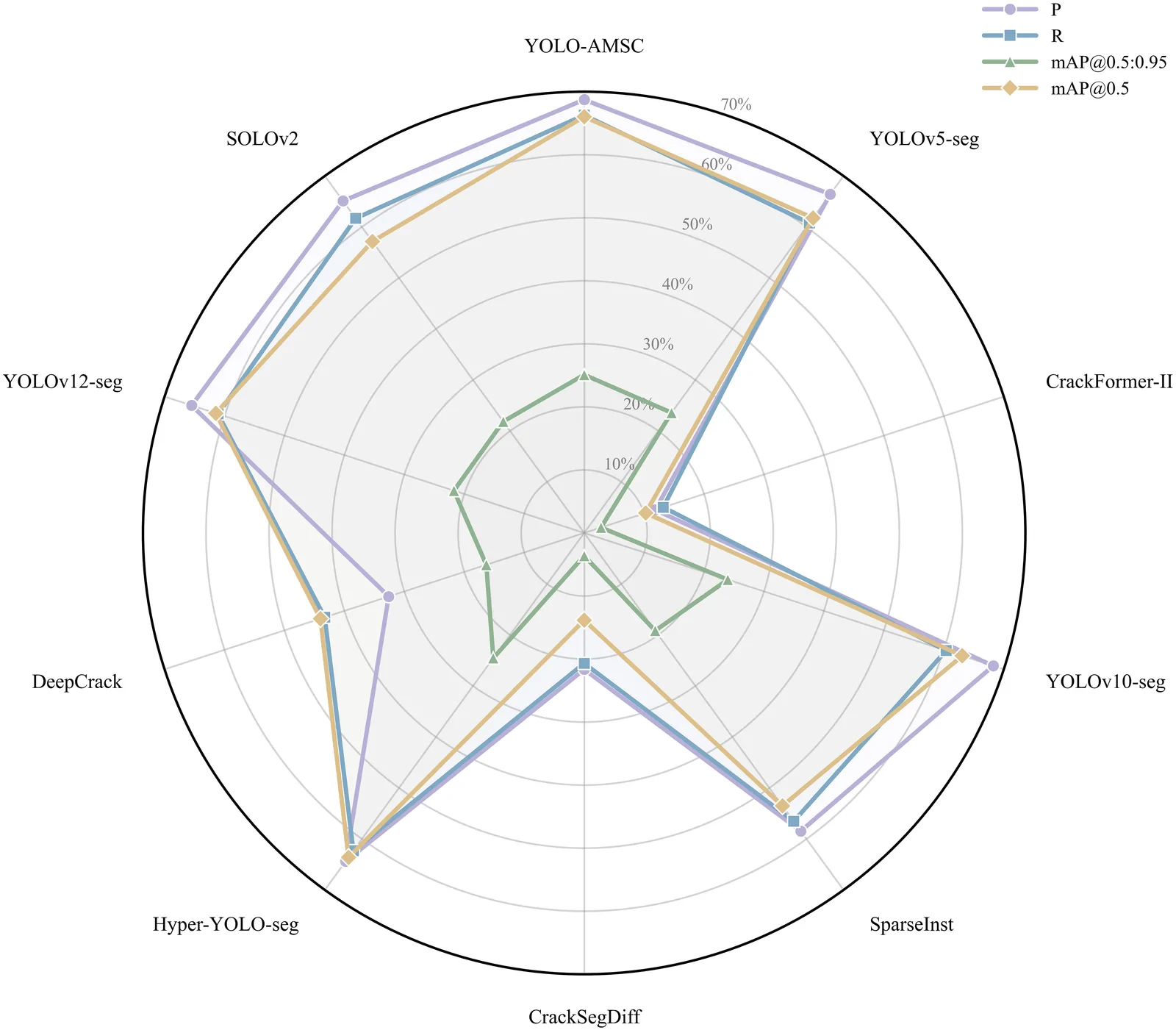
Comparison of segmentation performance among different models on a dataset of cracks in the mattic epipedon of alpine meadows.

The performance of the YOLO-AMSC network in crack segmentation in the mattic epipedon of alpine meadows was evaluated by preparing training datasets for alpine meadow cracks under uniform conditions with YOLOv5n-seg, YOLOv10n-seg, YOLO-hyper-seg, YOLOv12n-seg, SOLOv2, SparseInst, DeepCrack, CrackFormer-II, and CrackSegDiff. Subsequently, the performance of these ten segmentation models was evaluated using the test set. This study rigorously compared YOLO-AMSC with nine mainstream instance segmentation networks quantitatively. The experimental results show that YOLO-AMSC outperforms the other models across four key evaluation metrics. Specifically, it achieves a recall rate of 66.29%, which is 3.99% higher than the second-best model, hyper-yolo-seg, addressing the perception bottleneck observed in the conventional general-purpose model SparseInst of 56.54% under minor occlusion gaps. Notably, the crack-specific models DeepCrack, CrackFormer-II, and CrackSegDiff exhibit limited cross-domain generalization in complex natural backgrounds of alpine meadows, with mAP50–95 values of only 16.33%, 2.81%, and 3.63%, respectively. Additionally, by adaptively enhancing feature channels, YOLO-AMSC achieves a precision of 68.73%, surpassing both YOLOv12n-seg of 65.48% and the classic SOLOv2 of 65.10%, effectively reducing false positives in complex natural backgrounds. Significantly, YOLO-AMSC outperforms YOLOv12n-seg by 3.37% on the rigorous metric mAP50-95, reflecting superior pixel-level edge detection. This underscores the robustness of the YOLO-AMSC algorithm designed for crack analysis in the mattic epipedon of alpine meadows, enabling the precise measurement of crack width and the quantification of the degree of degradation. **Table 3** presents segmentation outcomes for various models, while **Figure 9** visually depicts performance variations among the models across diverse metrics.

**Table 3:** Comparative tests.

ID	Model	P	R	mAP50	mAP50-95
1	yolov5n-seg	66.42%	60.76%	61.76%	23.58%
2	yolov10n-seg	68.23%	60.38%	63.07%	23.96%
3	yolo-hyper-seg	64.38%	62.30%	63.60%	24.53%
4	yolov12n-seg	65.48%	61.08%	61.45%	21.73%
5	SOLOv2	65.10%	61.69%	57.17%	21.85%
6	SparseInst	58.46%	56.54%	53.52%	19.16%
7	DeepCrack	32.61%	43.28%	44.00%	16.33%
8	CrackFormer-II	11.86%	13.18%	10.26%	2.81%
9	CrackSegDiff	21.64%	20.68%	13.84%	3.63%
10	yolo-AMSC	68.73%	66.29%	66.03%	25.10%

### 4.3 Visualization results

**Figure 10:**
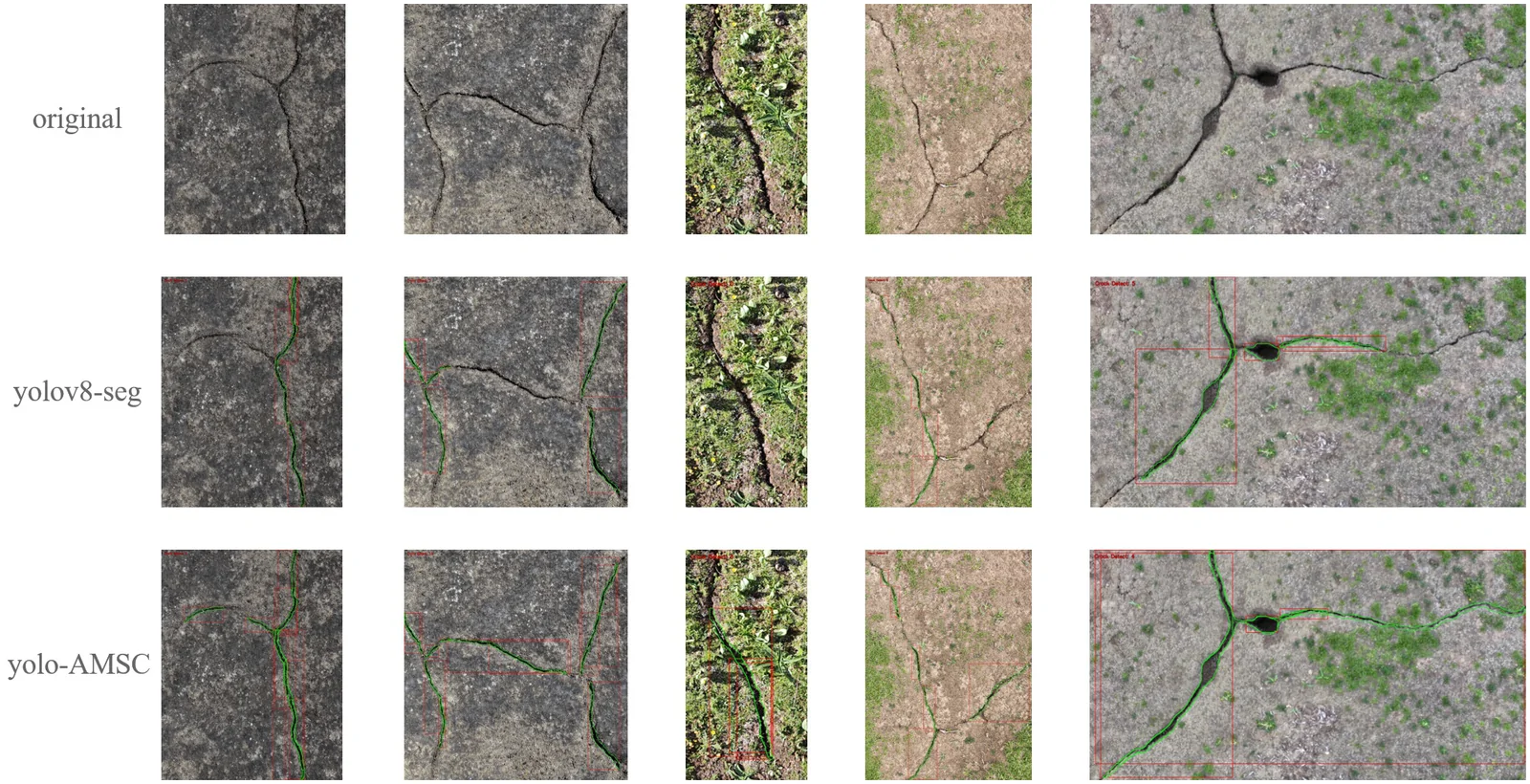
Visualization results for YOLOv8n and YOLO-AMSC across five image sets.

Visual comparison across the five representative samples in **Figure 10** reveals that YOLO-AMSC generally outperforms the baseline YOLOv8n in segmenting cracks in the mattic epipedon of alpine meadows. In scenarios characterized by poor illumination or dense vegetation obscuration, the baseline model exhibits conspicuous missed detections. Conversely, YOLO-AMSC effectively delineates the complete crack morphology, faithfully reproducing the intricate topological structures while maintaining exceptional continuity in its segmentation outcomes. Within the mattic epipedon of alpine meadows, factors such as vegetation textures, exposed soil, and shadows often induce interference, leading to inaccuracies in baseline models. YOLO-AMSC effectively mitigates these false alarms, demonstrating superior interference resistance. These findings underscore the precise spatial analysis enabled by the SDAF-Block and the adaptive anti-interference capabilities of the MLCA mechanism across the channel dimension, showcasing exceptional collaborative spatial-channel reconstruction. This approach successfully addresses the issues of missed detections and excessive false positives prevalent in baseline models, ensuring that YOLO-AMSC delivers consistently precise results for cracks in the mattic epipedon of alpine meadows in intricate ecological settings.

**Figure 11:**
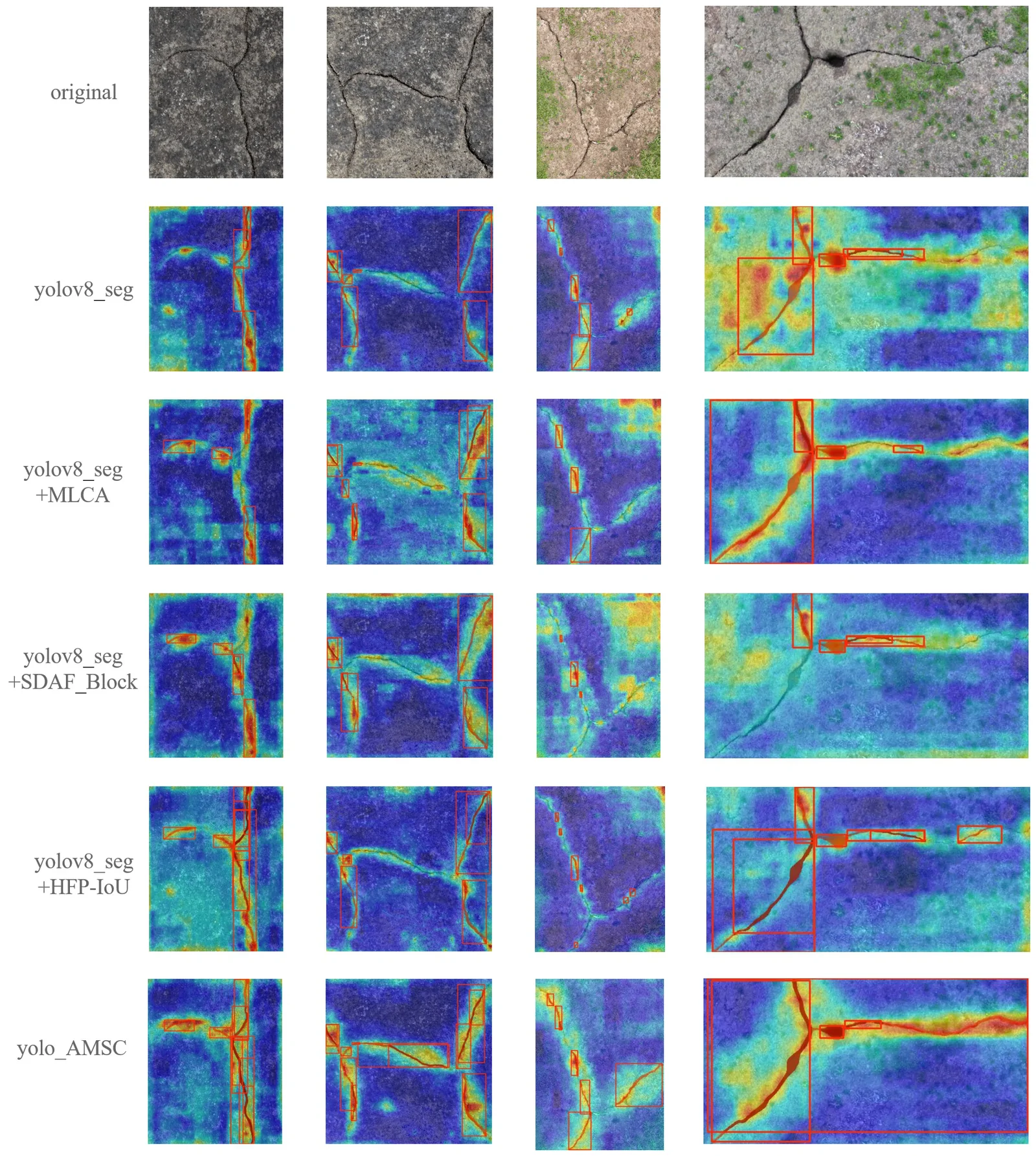
Heatmaps before and after the integration of the improved module.

Moreover, this research employs Gradient-weighted Class Activation Mapping (Grad-CAM) to visually scrutinize the network’s feature extraction process and generate representative heatmaps (Li et al., 2023a; Zhang et al., 2025a), as illustrated in **Figure 11**. This approach is applied to explore the impact of the enhancement module on the acquisition and depiction of desired features (Ren et al., 2026; Wang et al., 2025b; Li et al., 2026b). Specifically, for segmenting cracks in the mattic epipedon of alpine meadows, YOLOv8n exhibits fragmented activation areas. In contrast, the SDAF-Block excels in fine-grained spatial preservation and topological reconstruction; the MLCA mechanism strengthens background suppression and channel purification; and HFP-IoU imposes rigorous geometric boundary constraints. Collectively, these components enable a more precise segmentation of cracks in the mattic epipedon of alpine meadows, underscoring the scientific validity and rationale of YOLO-AMSC in addressing complex unstructured segmentation challenges.

### 4.4 Quantitative measurement and error analysis of crack width

Precise width measurement is paramount for the automated degradation assessment of cracks in the mattic epipedon of alpine meadows. While conventional approaches are susceptible to blurred boundaries and topological discontinuities, the skeletal geometric constraint-based technique proposed here relies on high-fidelity segmentation masks. To evaluate the practical utility of YOLO-AMSC for physical quantification, comparative experiments were conducted using actual width measurements. Representative samples were selected from crack segments with clear boundaries, continuous skeletons, and reliable correspondence between the UAV orthomosaic and field measurement locations. YOLO-AMSC was used to generate accurate crack boundaries and topological skeletons for these samples. By combining pixel-scale calibration with the intersections between orthogonal normal vectors and crack boundaries, the physical width at each skeleton node was calculated. For manual validation, crack widths were repeatedly measured using a DL91200 high-precision electronic digital caliper placed approximately perpendicular to the crack orientation, and the mean value was used as the reference width to reduce uncertainties caused by caliper reading, placement direction, boundary irregularity, and pixel-scale calibration. Detailed error statistics are summarized in **Table 4**.

**Table 4:** Calculated crack widths in the mattic epipedon of alpine meadows.

ID	Pixel width (px)	Actual width (cm)	Manual measurement (cm)	Absolute error (cm)	Relative measurement error (%)
1	420.05	1.7642	1.6716	0.0926	5.25%
2	307.51	1.2915	1.4066	0.1151	8.91%
3	389.14	1.6344	1.6133	0.0211	1.29%
4	289.85	1.2174	1.2808	0.0634	5.21%
5	388.86	1.6332	1.683	0.0498	3.05%
6	336.27	1.4123	1.3059	0.1064	7.53%

In this experiment, high-fidelity segmentation masks were integrated with an analytical algorithm grounded in skeletal geometric constraints to conduct comparative measurements of physical crack widths. Quantitative data reveal that this end-to-end framework exhibits high geometric accuracy, with the absolute error between calculated widths and manual field measurements ranging from 0.0211 cm to 0.1151 cm, while the relative error remains below 10%. YOLO-AMSC demonstrates exceptional feature extraction capabilities, effectively mitigating the grid-induced cumulative errors inherent in traditional discrete pixel statistics. Consequently, this approach provides dependable quantitative data to facilitate automated and comprehensive assessments of degradation levels for cracks in the mattic epipedon of alpine meadows at a regional scale.

To evaluate the skeleton-based geometric constraint for crack width estimation, we conducted robustness experiments under non-ideal imaging conditions, including varying contrast, motion blur, occlusion, and different signal-to-noise ratios. In these experiments, the width mean absolute error (MAE) and relative width error (RWE) were calculated using the algorithm-estimated width under normal imaging conditions as the reference, so as to quantify the width deviation induced by non-ideal imaging conditions. The results show that the proposed method has a certain degree of robustness to mild intensity variations and mild-to-moderate occlusion. Under γ = 1.5, γ = 2.0, and occlusion ratios of 5%–15%, the RWE remains relatively low, and the failure rate is also limited. However, motion blur has the most significant impact on skeleton-based width estimation. When the blur kernel increases from K = 5 to K = 21, the width MAE increases from approximately 0.010 cm to 0.033 cm, the RWE sharply increases from approximately 35% to 113%, and the failure rate increases from approximately 20% to 70%. These results indicate that severe blur reduces the visibility of crack boundaries, causes fragmentation of the segmentation masks, and further leads to skeleton displacement, increased spurious branches, and failure of width measurement.

**Figure 12:**
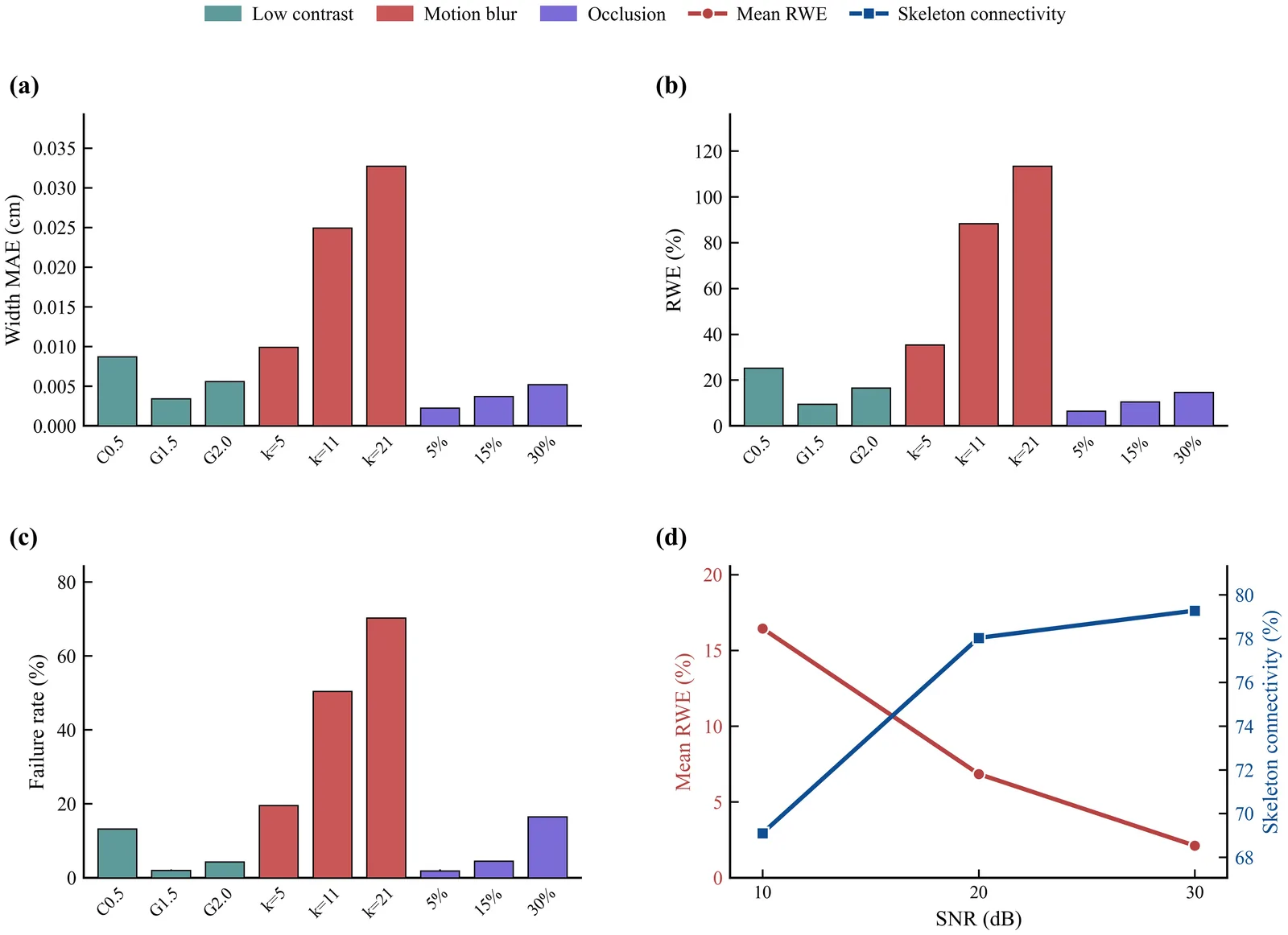
Robustness analysis of skeleton-based crack width estimation under image degradation: **(a)** width mean absolute error (MAE); **(b)** relative width error (RWE); **(c)** measurement failure rate; and **(d)** mean RWE and skeleton connectivity under different signal-to-noise ratios (SNRs).

**Figure 13:**
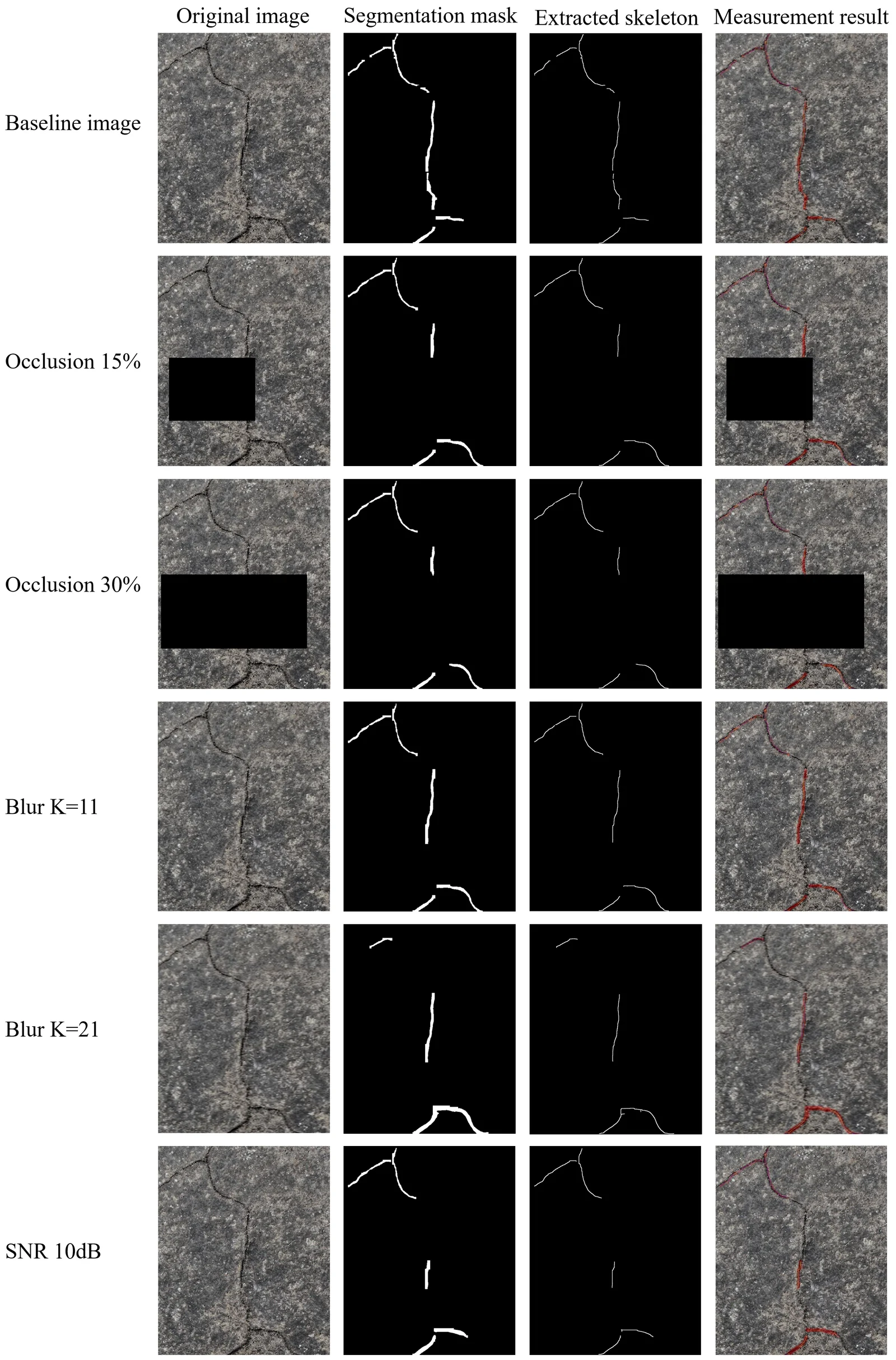
Failure cases of skeleton-based crack width estimation under mask fragmentation.

The SNR results in **Figure 12** further reveal the intrinsic relationship between skeleton topological continuity and width estimation reliability. When the SNR increases from 10 dB to 30 dB, the mean RWE decreases from approximately 16.5% to 2.1%, while the skeleton connectivity rate increases from approximately 69% to 79%, indicating that reduced image noise helps preserve the continuity of the main crack skeleton. Combined with the failure cases shown in **Figure 13**, when the occlusion ratio reaches 30% or the motion blur kernel increases to K = 21, the crack mask and skeleton topology are clearly disrupted. In these cases, the method does not force width output; instead, they are identified as measurement failure cases. Based on these results, the operational boundary of the proposed framework is defined as follows: the method is reliable when image quality is moderate to high, crack boundaries remain distinguishable, SNR is no lower than 20 dB, and the occlusion ratio does not exceed 15%. In contrast, under low SNR, occlusion close to 30%, or severe motion blur, especially when K > 11, the results are regarded as low-reliability measurements.

## 5 Discussion

This study enhances segmentation accuracy for cracks in the mattic epipedon of alpine meadows to support ecosystem degradation assessment via the developed YOLO-AMSC network. Fine-grained cracks in alpine meadows are typically narrow, low-contrast, curvilinear, and discontinuous, and are easily obscured by vegetation, exposed roots, soil particles, and shadows. To address these imaging challenges, YOLO-AMSC follows a design logic of “spatial topology preservation–contextual continuity recovery–channel-wise background suppression.” The SDAF-Block preserves crack topology and restores fragmented continuity under occlusion, providing a stable basis for skeleton extraction and width measurement. MLCA enhances crack-sensitive channels while suppressing crack-like responses from vegetation, soil particles, and shadows. HFP-IoU further strengthens geometric constraints on elongated boundaries, reducing boundary deviation and producing more compact pixel-level masks. Overfitting in unstructured vegetation backgrounds is mitigated by the compact YOLOv8n-seg backbone, cross-stage partial feature reuse, channel-shared recalibration in MLCA, training weight decay, and the multi-regional dataset, encouraging the model to learn physically meaningful crack morphology rather than site-specific vegetation textures or illumination patterns. Visualization via activation maps validates this rationale: whereas the YOLOv8n-seg baseline exhibits scattered attention, YOLO-AMSC heatmaps demonstrate intense focus following the crack’s morphology, ensuring object-centric segmentation amidst complex backgrounds.

The crack-width thresholds of 0.6 cm and 3.1 cm should be interpreted as morphological warning values under the alpine meadow conditions of the present study area, rather than as universal ecological degradation boundaries. Although crack width provides a direct and quantifiable indicator for UAV-based rapid monitoring, alpine meadow degradation is jointly regulated by vegetation–root–soil–water–geomorphology interactions. Previous studies have shown that crack length, width, depth, and length density in alpine meadows are closely associated with root length density. With increasing degradation, vegetation cover decreases, roots accumulate in the surface layer, and crack morphology gradually evolves from linear cracks to dendritic and polygonal networks (Wu et al., 2024b). Further evidence indicates that the shift in plant communities from fine-rooted grasses and sedges to coarse-rooted forbs weakens root–soil reinforcement in alpine meadows, while root length density serves as a key factor controlling crack propagation and morphological evolution. Wetting–drying and freeze–thaw stresses can further accelerate crack expansion and promote a self-reinforcing degradation process (Wu et al., 2026). From a hydro-mechanical perspective, surface cracking begins when the water content of alpine meadow root–soil composites decreases to 30.00%–40.00% and matric suction increases to 50.00–100.00 kPa; with further drying, linear cracks may develop into dendritic and polygonal patterns (Zhang et al., 2025b). Therefore, under the investigated site conditions, the 0.6 cm threshold can be regarded as an early morphological signal of increasing crack connectivity, whereas the 3.1 cm threshold indicates severe fragmentation of the mattic epipedon. These thresholds are more appropriate as regional warning indicators for alpine meadows with similar vegetation, soil moisture, root–soil structure, grazing history, and geomorphological conditions.

Precise segmentation masks are indispensable for accurate physical width quantification. Results from the width estimation approach, based on skeletal geometric constraints, demonstrate that the absolute error between computed and manual measurements is consistently within a few millimeters, with the relative error maintained below 10%. Leveraging the established end-to-end framework, we performed automated scanning and evaluation of a large-scale dataset comprising 5,305 images of cracks in the mattic epipedon of alpine meadows. Subsequently, quantitative classification was executed according to the Assessment Standard for Degradation Status of Alpine Mattic Epipedons (DB63/T 1413–2015), followed by a comprehensive analysis of multi-parameter correlation data. Although the dataset covers six representative sites in Menyuan, Dari, and Maqin counties, the current results do not constitute strict external validation in entirely unseen regions. Differences in vegetation composition, soil texture, illumination conditions, acquisition season, and sensor characteristics may introduce domain shifts. Active domain adaptation based on regional consistency and predictive certainty provides a feasible strategy for transferring segmentation models to new regions with limited target-region annotations (Guo et al., 2026). Future work will conduct region-based validation and independent external-region validation to further assess and improve the cross-regional applicability of YOLO-AMSC.

### 5.1 A nonlinear quadratic relationship between crack width and crack area ratio

**Figure 14:**
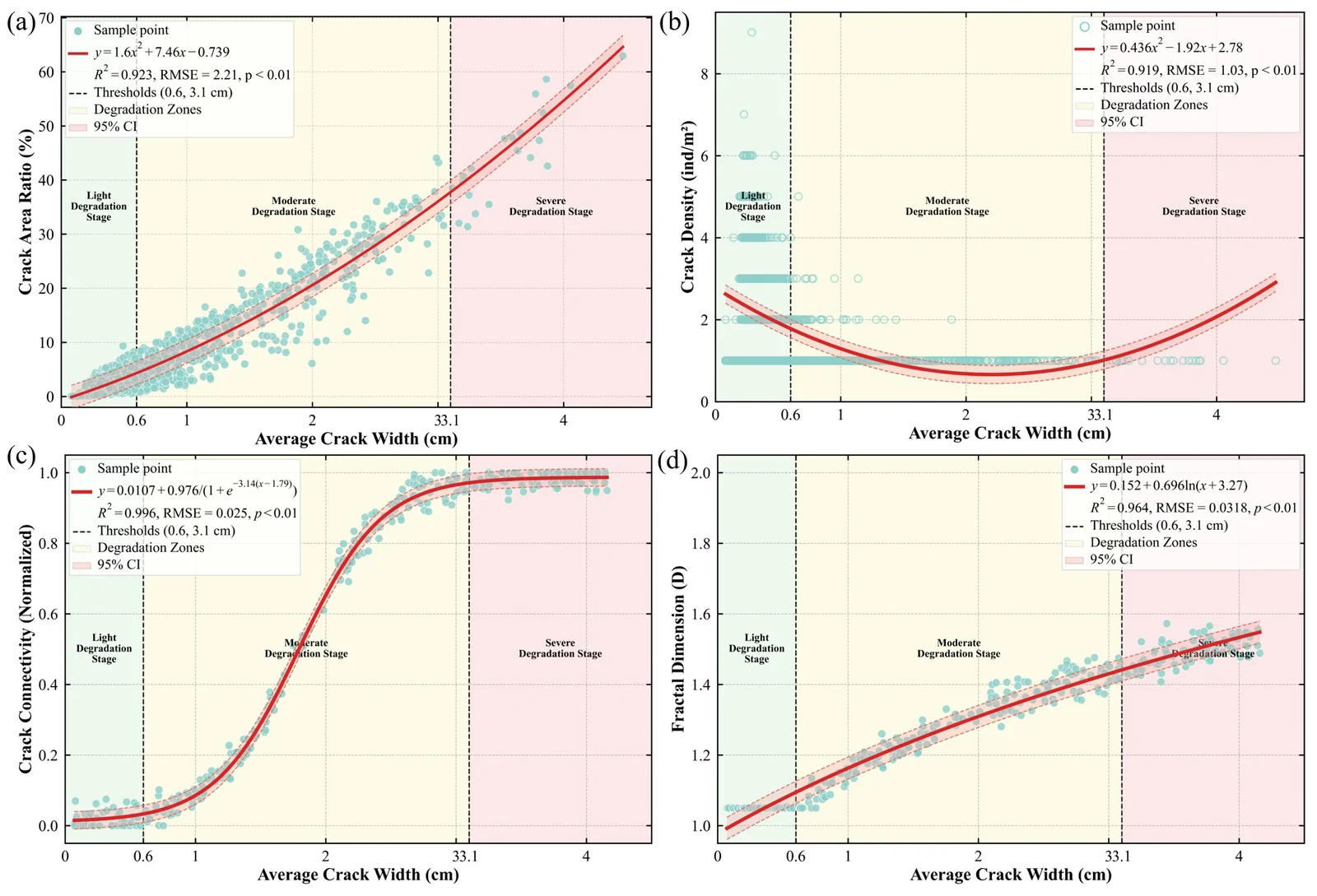
Non-linear fitting relationships between the average crack width of mattic epipedon of alpine meadows and multidimensional geometric indices: **(A)** relationship between average crack width and crack area ratio; **(B)** relationship between average crack width and crack density; **(C)** relationship between average crack width and crack connectivity; **(D)** relationship between average crack width and crack fractal dimension.

Regression analysis in **Figure 14a** uncovers a significant non-linear relationship between mean crack width and the crack area ratio (CAR) in the mattic epipedon of alpine meadows. The expansion of exposed surface area is not merely a consequence of linear widening; rather, a subtle increase in crack width can trigger a disproportionate deterioration in surface habitat area. This underscores the role of crack width as a sensitive early-warning indicator for ecosystems in the mattic epipedon of alpine meadows. Furthermore, these findings validate the scientific feasibility of accurately predicting two-dimensional habitat loss by utilizing a one-dimensional linear indicator that is easily monitored in the field.

The non-linear, heterogeneous expansion pattern observed in the mattic epipedon of alpine meadows signifies the physically induced, phased disintegration mechanism of the grass mat layer, highlighting the critical threshold where the system shifts from latent degradation to catastrophic fragmentation. By correlating this physical evolution process with the nonlinear quadratic response of CAR to average crack width, we divided the process into three stages, each exhibiting unique force dynamics and specific degrees of degradation:At the mild degradation stage (<0.6 cm), mechanical anchorage and the tensile toughness of the root-soil complex impose strict constraints. The system operates within the “structural resilience zone,” with the CAR maintained at a low level of<8%. Upon reaching the moderate degradation stage (0.6–3.1 cm), that is the critical region where the curve undergoes a geometric inflection point, The mechanical tolerance threshold of the root complex was exceeded, triggering a catastrophic positive-feedback collapse mechanism, resulting in a super-linear, explosive increase in CAR to 35–40%. Additionally, the scatter plot demonstrates a significant rise in variance, reflecting the sudden increase in the connectivity and heterogeneity of the crack network at this stage. As the deterioration progressed to an advanced stage (>3.1 cm), the fitted curve exhibited a steep slope, with the CAR surpassing 40% and nearing 65%. This indicates that the landscape has undergone a stable-state shift and substrate inversion, irreversibly transitioning from a vegetation-dominated state to a crack-dominated bare-soil state, with a near-complete loss of ecosystem service functions.

### 5.2 Nonlinear U-shaped coupling mechanism between mean crack width and density

Analysis of **Figure 14b** reveals a distinctive non-linear “U-shaped” relationship between the average width and density of cracks in the mattic epipedon of alpine meadows, signifying a transition in soil failure mechanisms from diffuse damage to structural collapse. Curve fitting delineates the progression of crack formation through phases of high-density nucleation, followed by density reduction via merging, and a potential secondary phase of fragmentation rebound. This study provides deep insights into the irreversible dynamic succession of alpine meadow degradation. It demonstrates that internal soil stress release shifts as crack width increases: transitioning from diffuse micro-fracturing in the initial stages to localized primary fracturing in the middle stage, and ultimately to the irreversible structural fragmentation characteristic of the advanced degradation phase.

Adhering to the Assessment Standard for Degradation Status of Alpine Mattic Epipedons (DB63/T 1413–2015), two width thresholds of 0.6 cm and 3.1 cm are identified to categorize three degradation stages and elucidate the morphological trade-off mechanism. In the mild degradation stage where the average width remains below 0.6 cm, mechanical constraints from the root-soil complex’s tensile toughness prevail. Stress is dissipated through the formation of numerous discrete microcracks with a density exceeding 4 ind/m^2^. During the moderate degradation stage between 0.6 cm and 3.1 cm, adjacent microcracks coalesce. Guided by the principle of energy minimization, the system prioritizes primary crack propagation, exhibiting a trade-off where crack quantity decreases as individual widths increase. This marks a transition from diffuse microdamage to linked macroscopic pathways. Finally, in the severe degradation stage exceeding 3.1 cm, macroscopic cracks partition the soil into disconnected blocks. Secondary fracturing then initiates hierarchical fragmentation and granulation, causing a resurgence in crack density and signifying the total disintegration of the meadow soil’s structural matrix.

### 5.3 Identifying the tipping point of degradation via the sigmoid response of crack connectivity

Regression analysis in **Figure 14c** uncovers a nonlinear positive relationship between average crack width and connectivity, leading to the development of a sigmoid topological response model. Empirical findings suggest that connectivity evolves through three discernible phases: lagging accumulation, exponential surge, and saturated stability. This delineates the topological threshold where soil structure shifts from equilibrium to instability. By leveraging the features of the initial lag zone, a mean crack width of 0.6 cm is established as the key safety alert threshold for disturbance resilience, denoting the conclusion of the latent period of degradation. Crack widths below 0.6 cm correspond to connectivity persisting at a markedly low level of less than 0.1 with near-zero growth rates. From an ecological mechanics standpoint, this phase represents the resilience recovery area where the dense root mat exerts robust mechanical anchoring to constrain fissure enlargement. During this period, fissures remain spatially discrete and fail to form a functional hydrologically linked network. Consequently, runoff occurs primarily as sheet flow without established dominant routes, and 0.6 cm signifies the crucial threshold for preserving structural stability.

Within the steepest section of the Sigmoid model, spanning from 0.6 cm to 3.1 cm, the system undergoes a critical phase transition and positive feedback instability. This range establishes the topological foundation for the golden window of degradation management. Data suggest that once the average width surpasses the physical threshold of 0.6 cm, the mechanical anchoring of the soil-root complex fails. Consequently, connectivity experiences an explosive exponential increase with even minor increments in width, rising above 0.9 and indicating that the system has crossed the topological seepage threshold. During this phase, previously isolated micro-cracks merge extensively, initiating a positive feedback loop where widening cracks enhance connectivity and moisture retention, thereby intensifying erosion and freeze-thaw damage. These interconnected cracks then serve as preferential pathways for soil and water loss, leading to a rapid deterioration of hydrological regulation and driving the system toward irreversible collapse. Thus, if the 0.6 cm anchoring threshold is breached, this non-linear transition zone becomes the critical range for targeted scientific interventions to restore ecosystem stability.

### 5.4 Nonlinear transition of mattic epipedon of alpine meadows cracks from euclidean to fractal geometry

Analysis of **Figure 14d** shows a logarithmic relationship between the average crack width in the mattic epipedon of alpine meadows and the fractal dimension. This relationship quantifies how spatial heterogeneity on the soil surface escalates significantly with increasing degradation. The findings from the experiments suggest that the development of crack patterns does not follow a linear or uniform progression. Instead, it displays clear non-linear traits, with the fractal dimension transitioning from approximately 1.0, typical of a straight Euclidean line, to 1.55, shaping an intricate network structure. This quantitative relationship suggests that, as alpine meadows degrade, the morphology of cracks transitions from simple one-dimensional linear structures to complex two-dimensional network structures. This transition offers the most intuitive and scientifically grounded geometric indicators for developing an early-warning and classification management system for alpine ecosystems utilizing deep learning.

This study identifies three dynamic stages in the evolution of soil matrix morphology from Euclidean geometry to fractal structures, grounded in two critical width thresholds of 0.6 cm and 3.1 cm. In the initial mild degradation phase where average widths remain below 0.6 cm, the dense root mat exerts significant mechanical traction and constraints. Cracks primarily manifest as tensile fissures induced by freeze-thaw cycles, displaying regular shapes with smooth edges and minimal branching. The fractal dimension is concentrated between 1.0 and 1.1, demonstrating Euclidean linear properties typical of a morphologically stable phase. Upon entering the moderate degradation stage between 0.6 cm and 3.1 cm, the system reaches a geometric inflection point where the mechanical tolerance of the root complex is surpassed, triggering a positive feedback collapse. Rather than mere widening, cracks undergo lateral erosion and secondary branching, resulting in irregular, tortuous edges with self-similar topological characteristics. The fractal dimension escalates from 1.1 to approximately 1.45, signifying a non-linear phase transition from Euclidean geometry to a fractal structure. Consequently, the soil surface fragments further, leading to an exponential increase in exposed area vulnerable to wind and water erosion.

## 6 Conclusions

To resolve unstructured perception challenges in the mattic epipedon of alpine meadows, this research validates an end-to-end framework for precise crack segmentation and quantification. We developed YOLO-AMSC, utilizing the SDAF-Block and MLCA mechanisms to preserve fine crack features amidst complex backgrounds. Ablation and comparative analyses show that YOLO-AMSC achieves 68.73% precision, 66.29% recall, and 66.03% mAP50. Notably, the inclusion of HFP-IoU improves recall by 6.43% and mAP50 by 3.40% over the YOLOv8n-seg baseline. By effectively reducing false negatives from vegetation interference while sustaining an inference speed of 218.52 frames per second, the proposed model provides a robust solution for high-precision, real-time perception during large-scale drone-based ecological monitoring.

Utilizing high-fidelity segmentation masks, we developed an analytical algorithm based on skeletal geometric constraints to quantify crack widths. This approach converts discrete pixel signals into continuous geometric curves. By applying three-point interpolation to solve orthogonal normal vector equations at each skeletal node, it eliminates cumulative gridding errors typical of discrete pixel statistics. Field validation confirms the framework’s high geometric fitting accuracy, with absolute errors between calculated and manual measurements stay within 10%. Consequently, this system provides a reliable source of morphological data for automated, width-based degradation diagnostics in the mattic epipedon of alpine meadows.

By integrating high-precision pixel-level sensing with a geometric quantification framework, this study investigates the systematic dynamic mechanisms of crack development to bridge the gap between deep learning algorithms and macroecological diagnostics. Spatial parameter correlation analysis has, for the first time, quantitatively unveiled non-linear patterns of crack development using a comprehensive dataset. These include an allometric growth model linking crack width and habitat loss, a non-linear U-shaped evolutionary coupling, a sigmoid phase transition indicative of topological connectivity, and a significant geometric shift from Euclidean linear formations to fractal networks. Furthermore, this system adheres to the Assessment Standard for Degradation Status of Alpine Mattic Epipedons (DB63/T 1413–2015). Through data-fitted curves, it rigorously delineates the ecological significance of the 0.6 cm disturbance resistance threshold and the 3.1 cm irreversible structural collapse threshold. In summary, this research innovates deep learning models for complex alpine meadows and offers a reliable, deployable solution for non-destructive dynamic monitoring and identifying optimal intervention windows.

## Data Availability

The datasets generated and analyzed during the current study are available from the corresponding author on reasonable request. Requests to access the datasets should be directed to Haili Zhu qdzhuhaili@163.com.
